# A remanufacturing supply chain network with differentiated new and remanufactured products considering consumer preference, production capacity constraint and government regulation

**DOI:** 10.1371/journal.pone.0289349

**Published:** 2023-08-10

**Authors:** Xuemei Zhang, Gengui Zhou, Jian Cao, Jiansha Lu

**Affiliations:** 1 School of Mechanical Engineering, Zhejiang University of Technology, Hangzhou, China; 2 School of Management, Zhejiang University of Technology, Hangzhou, China; 3 Center for Global & Regional Environmental Research, The University of Iowa, Iowa City, Iowa, United States of America; Wuhan Textile University, CHINA

## Abstract

Remanufacturing is a sustainable product reutilization strategy to realize responsible consumption and production. However, remanufacturing practice can be subject to deficient consumer perception, production capacity constraint, uncertain demand and government intervention. This paper considers outsourced remanufacturing mode to establish and investigate a remanufacturing supply chain (RSC) network consisting of multiple manufacturers, retailers and remanufacturers. Optimality conditions with RSC network members are derived utilizing variation inequality. Numerical examples based on data accumulated from a remanufacturing enterprise and questionnaire survey on consumer perception for remanufactured products, are presented to put the proposed model into practice. Influences of product heterogeneity (new and remanufactured products), consumer preference, production capacity constraint, product price competitiveness, market demand fluctuation, and government financial measures on RSC network production and pricing strategies are investigated through numerical analyses. Analytical results indicate that pricing for the remanufactured product would be equivalent to that of the new product when consumers value the remanufactured product at a relatively high level, however an excessive penchant for the remanufactured product is less profitable especially from the perspective of the remanufacturer. In general, an upper-middle level of consumer preference for the remanufactured product is the most favourable scenario. When remanufacturing industry is just emerging, a high tax will be imposed on the manufacturer to accumulate remanufacturing subsidy for the government to achieve its balanced budget. As the remanufacturing industry develops and consumer environmental awareness enhances, it is especially essential for the government to establish a levy-subsidy mechanism to maintain sustainable progress of the industry. Combining main conclusions with the background of Chinese remanufacturing industry, managerial implications are provided from respective perspectives of enterprises’ remanufacturing operation, government financial regulation, and consumer awareness enhancement. The analyses and results are especially relevant as a reference for remanufacturing decision-makings as well as government financial regulations, thus enhancing production sustainability as well as environmental benefits.

## Introduction

Resource depletion and environmental deterioration have given rise to green legislation (e.g. tax and subsidy policies) to hold manufacturers accountable for their operations [1, 2]. Remanufacturing as a specific type of eco-efficiency technology, which recovers, processes, and sells like-new versions of obsolete products, is playing a paramount role in environmentally conscious industrial efforts [3, 4]. A remanufactured product can match the quality and performance of a new product, while remanufacturing the used product reduces both resource depletion and negative environmental externalities [5–9].

Many governments recognize that remanufacturing of used products at their end-of-life (EOL) can contribute to environmental protection and resource conservation aims. They have implemented legislation requiring firms to collect EOL products, such as the EU’s Waste Electrical and Electronic Equipment directive, and Extended Producer Responsibility principal, the US’s Electronics Recycling laws, and Japan’s Waste Treatment Mechanism. To comply with these regulatory schemes, firms face additional challenges to adjust their existing production and pricing strategies, responding either proactively or reactively to the implementation of EOL product recovery strategies, such as remanufacturing. Corporates, like Hewlett-Packard, Kodak, IBM, and Fuji, are proactively designing products to accommodate future remanufacturing. The used products of Kodak, for example, have high remanufacturability– 70% of the core components can be recycled and remanufactured [10, 11]. Xerox recycles reusable cartridges from consumers to produce a new copier, thereby helping to cut ~65% production costs through remanufacturing [12]. In practice, Firms like GE Aviation, Mercedes-Benz, and Xerox, to name a few, have established self-remanufacturing or outsourced remanufacturing systems. In the latter case, a third-party remanufacturer (3PR) performs the remanufacturing activities, while the original equipment manufacturer (OEM) retains responsibility for marketing the remanufactured product. Many OEMs in the US and Europe prefer to outsource remanufacturing operations. For instance, Land Rover and Caterpillar have signed a contract by which Caterpillar Remanufacturing Services is entrusted as Land Rover’s remanufacturing services provider [13, 14].

Manufacturing/remanufacturing decisions are made even more complicated given the heterogeneity in consumers’ attitudes toward the type of products (new or remanufactured). Most consumers typically value a remanufactured product less due to quality and reliability concerns, yet increasing numbers of consumers who care about environmental issues have presented market opportunities and may be a pivotal motive for remanufacturing in many instances. An EU survey justifies the latter trend: approximately 8 out of 10 citizens value the environmental effect of a product as a critical criterion when purchasing differentiated products [15]. The surge in consumer awareness and demand for greater sustainability has intensified environmental considerations throughout remanufacturing operations. This makes it imperative for firms to incorporate product heterogeneity, consumer preference, and market demand uncertainty into their manufacturing/remanufacturing strategies so as to pursue potential benefits while avoiding negative economic and social consequences.

Under mounting pressure from the public and facing more stringent regulations, firms have come to realize that the major way to reduce their burden is through their supply chain networks [16, 17]. Some researchers [18–20] have extended previous work to strategically model closed-loop supply chain (CLSC) networks. However, there are limited studies that address the complexity arising from product heterogeneity, consumer perception, and production capacity constraints. In addition, the internalization of environmental costs through financial instruments (tax and subsidy) makes the interrelations between new and remanufactured products and associated production and pricing strategies more intricate.

This study aims to provide a reference framework for remanufacturing supply chain (RSC) decision-making as well as government financial regulations. Considering production capacity constraint, an RSC network model with differentiated products and consumer preferences under government financial regulation is established. To demonstrate the practicality of the proposed model, we provide numerical examples for *numerical control machines*, a product category that is included in remanufacturing industry. The setup of government fiscal policy regarding tax and subsidy measures is elaborated according to the “*Swap the old for remanufacturing*” carried out by National Development and Reform Commission (NDRC), Ministry of Finance (MOF) and Ministry of Industry and Information Technology (MIIT) of Chinese government. Using the data and expert input from the remanufacturing industry, we derive the equilibrium solutions of the RSC network through variational inequality method, and capture the influences of consumer preference variance, production capacity constraint, product price competitiveness, market demand fluctuation, and fiscal tools on RSC network strategies. The proposed model can be flexibly applied to various directions for further investigation and implications, if particular parameters or parameter ranges are provided. These implications could prove valuable to practitioners and authorities for the promotion of remanufacturing industry.

The rest of this paper is organized as follows. In Literature review, an overview of the literature is presented to provide support for and positioning of this study. Then the RSC network model is established. We derive the supply chain network governing equilibrium conditions and provide the variational inequality formulations. A practical case and numerical examples are provided and discussed in the following. Conclusions and implications summarizes the study with corresponding managerial implications, and provides directions for future research.

## Literature review

We limit our review to three streams of literature closely related to the topic of interest. The first body of literature pertinent to our study is that on CLSC decision-making considering consumer perceptions of remanufacturing and remanufactured products. Remanufacturing is regarded as the cornerstone of sustainable supply chain operations yet has remained a largely untapped opportunity for enhancing supply chain productivity because of poor consumer perceptions of remanufactured products [21, 22]. In recent years, an increasing body of research on CLSC strategies for remanufacturing considering consumer environmental awareness and behaviour has developed. The confusion and lack of understanding, along with the negative perceptions of remanufactured products, pose a vexing issue for remanufacturing-related activities [23, 24]. Wang et al. [25] stated that the manufacturers should focus on enhancing consumer intention to purchase remanufactured products in marketing of this kind of product. Zhou et al. [26] set up a game-theoretical model to examine the implications of consumer education upon a CLSC, figuring out that consumer education enlarges the proportion of consumers who are willing to pay for the remanufactured product. Meanwhile, consumers do not have consistent perceptions of the quality and associated risks of purchasing a remanufactured product [27, 28]. Abbey et al. [29] empirically investigated consumer perceptions of remanufactured products and concluded that green consumers typically found remanufactured products more attractive. Consumers who are very concerned about environmental problems tend to purchase remanufactured products in order to avoid the chemicals and toxins that new products may contain [30]. By and large, remanufacturing decisions considering consumer preference have been extensively studied in previous literature, however most studies are based on the assumption that pricing for the new product is higher than that for the remanufactured one, neglecting that consumers’ heightened environmental awareness has opened a new avenue for repricing differentiated products. The proposed model in this paper captures pricing strategies that change with consumer perception variance. Besides, most researches are considered in a single supply chain other than a network involving multiple supply chains. The proposed RSC network model seeks to fill this gap by including the complexity that simultaneously arises from product heterogeneity and supply chain network coopetition.

The second remanufacturing streams of literature related to our work concerns modelling the CLSC network utilizing the variational inequality method. Since consumers, regulations, and resource constraints have caused additional challenges for firms, they have come to realize that the major way to reduce their burden is through supply chain networks [31–33]. Based on the concept of equilibrium, Nagurney et al. [34], Nagurney and Toyasaki [35] first explored a variational inequality CLSC model consisting of manufacturers, retailers, and demand markets, with the inclusion of recycling. Yang et al. [36] expanded this work and integrated the reverse supply chain into the oligopolistic model, which includes manufacturers that produce homogeneous commodities from raw materials and reusable ones, and recovery centres that recycle used products. Since then, many authors have documented the development and application of equilibrium models on CLSC management using optimization theory, variational inequality, and network theory, among others. Qiang et al. [37] examined a CLSC network consisting of suppliers, retail outlets, manufacturers, and the demand market using variational inequality. Based on variational inequality and complementarity theory, Amin et al. [18] explored the optimal behaviour and equilibrium solutions of CLSC network members under carbon emission constraints. Saberi et al. [38] presented a competitive supply chain network model considering green technology investment options, which allowed firms to better manage production, inventory, shipment, and technology investment. Fu et al. [39] developed a coupled CLSC network model to analyse how market size and raw material costs impact CLSC network decisions and profits. In most of the literature concerning CLSC network models on remanufacturing, it overlooks the impacts of production capacity constraint and product heterogeneity on remanufacturing decisions. Meanwhile, the literature related to the demand market is still in its infancy relative to many other CLSC network domains. To address the research gap, we consider consumer preference variance, production capacity constraint, and utilize fuzzy demand, to capture product price competitiveness and market demand fluctuation, in modelling the significant uncertainties in demand for new and remanufactured products.

The third stream of research focuses on government interventions via tax and subsidy measures. Researchers have utilized regulatory policies for internalizing externalities, such as taxes or recovery subsidies, in a product remanufacturing supply chain model design. Taxation, along with the market mechanism pertinent to waste emissions, is one way of incorporating environmental costs into the supply chain [40]. Miao et al. [41] studied the optimal pricing and production strategies of manufacturers under a carbon tax policy and a cap-and-trade program, revealing that environmental benefits could be elevated with well-designed government regulations. Qiu and Jin [42] investigated the impact of environmental taxes on manufacturers’ remanufacturing decisions in three cases of a duopoly, suggesting policymakers to employ a stricter environmental tax policy. Government subsidy is another regulatory approach in the early stage of the remanufacturing industry, acting as a crucial stimulus to incentivize manufacturers/remanufacturers [43]. Considering government subsidy policy, Zhao et al. [44] examined the joint decision problem for price determination of remanufactured products and subsidy share between a remanufacturer and consumers. Zhang et al. [45] found that subsidy policy is superior to tax policy for remanufacturing, but may lead to heavy environmental burdens. Nie et al. [46] investigated the impact of government subsidy on remanufacturing profit and environmental performance, through a comparative analysis of a game theoretical model of two competing manufacturers. In general, the literature has ignored the need for a balanced budget of government when regulating remanufacturing activities via tax and subsidy measures, which is essential for the conducive support of government regulation. The proposed model in this paper filles the research gap by addressing the influences of government’s attempt to make ends meet.

To extend the research and provide additional implications for governments and managers from economic and environmental perspectives, this paper introduces an RSC network with differentiated new and remanufactured products. The model considers consumer preference discrepancy, production capacity constraint, product price competitiveness, demand uncertainty, as well as government involvement via fiscal instruments to achieve a balanced budget between manufacturing tax (levy imposed on a unit of new product) and remanufacturing subsidy (subsidy offered for a unit of remanufactured product). We derive the optimality conditions of members within the RSC network and establish the governing equilibrium conditions, which can be formulated as a variational inequality problem. Using real data of a remanufacturing enterprise and questionnaire survey on consumer preference for the remanufactured product, the impacts of consumer preference, production capacity constraint, product price competitiveness, demand fluctuation, and government intervention on RSC network strategies are investigated. Analytical results and managerial insights have significant ability to enhance RSC network benefits.

## The RSC network with differentiated products

**Fig 1:**
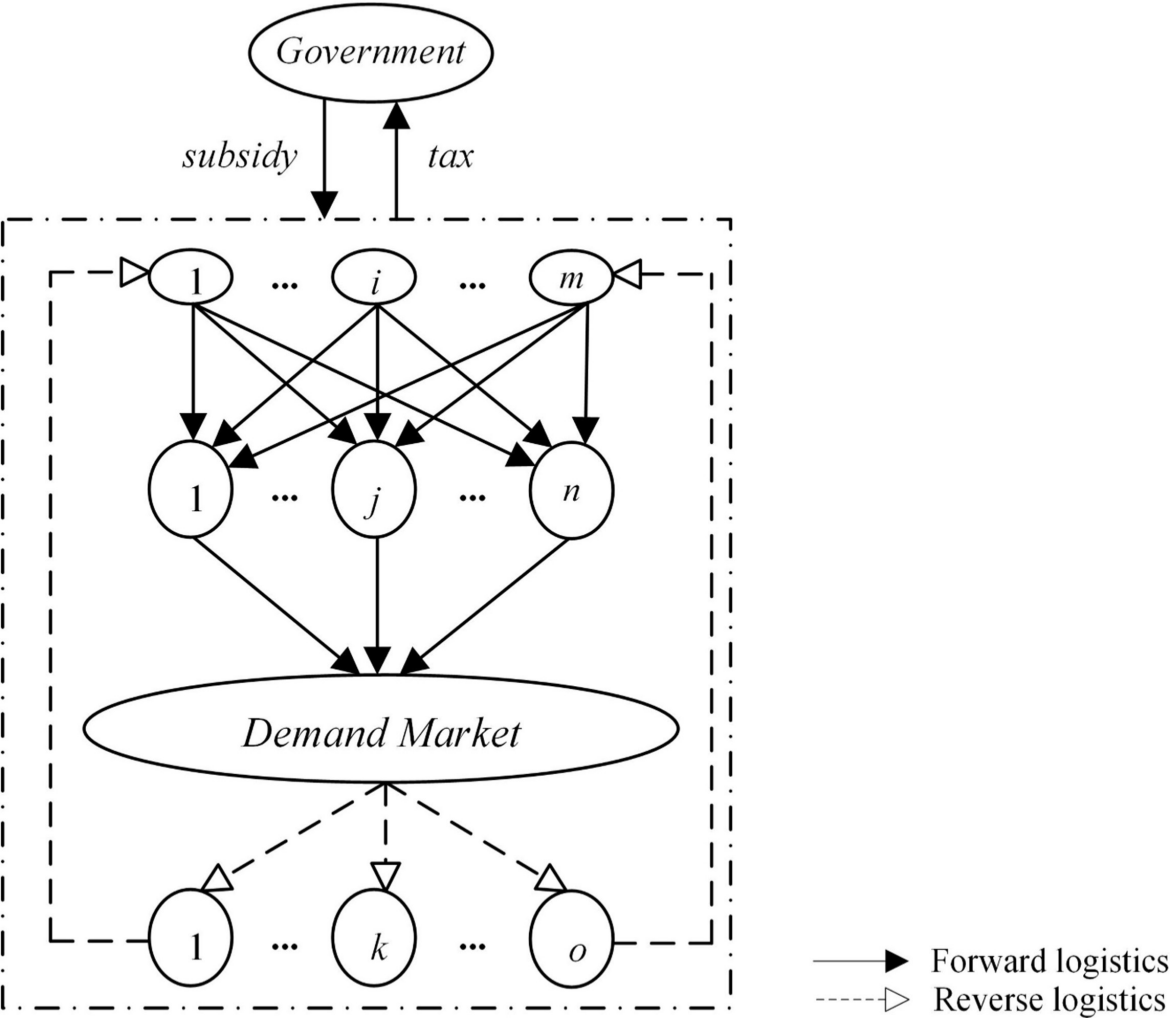
Model framework of the RSC network.

The RSC network consists of *m* OEMs (hereafter referred to as manufacturers), each one denoted by *i* (*i* = 1,⋯,*m*), *n* retailers, with a typical one denoted by *j* (*j* = 1,⋯,*n*), *o* 3PRs (hereafter referred to as remanufacturers), each one defined by *k* (*k* = 1,⋯,*o*), and one typical demand market (all notations are explained and listed in S1 Appendix, for convenience of reference and understanding). The manufacturers are involved in the production of the new product from raw materials and outsource remanufacturing operations to remanufacturers. The retailers sell both new and remanufactured products to the market. The new and remanufactured products are substitutable but distinct. Consumers in the demand market can return their used products to remanufacturers, which are entrusted by the manufacturers to collect and remanufacture EOL products. The government regulates RSC network operations via financial instruments based on its balanced budget, i.e. the remanufacturing subsidy provided for the remanufacturer is generated from the production tax imposed on the manufacturer. Fig 1 depicts the framework of the RSC network, where the solid line represents the product flow in the forward chain and the dashed line indicates the reverse chain.

### The behaviour of manufacturers and their optimality conditions

In the RSC network, *m* manufacturers compete in the production of a substitutable product, and each manufacturer *i* is assumed to have a production limit of $\overset{‐}{q}$ owing to resource and technical constraints. Let $q_{i}^{N}$ denote the production output of the new product made by manufacturer *i*, and group all $q_{i}^{N}$s into an *m*-dimensional column vector $Q_{m}^{N}$. The manufacturers make products with raw materials, purchase the remanufactured products from remanufacturers, and then sell both new and remanufactured products to retailers. Let $q_{ij}^{N}$ and $q_{ij}^{R}$ denote respective transaction quantities of new and remanufactured products between manufacturer *i* and retailer *j*. We group these variables into *mn*-dimensional column vectors $Q_{mn}^{N}$ and $Q_{mn}^{R}$, respectively. Let $q_{ki}^{R}$ denote the transaction quantity of remanufactured product between remanufacturer *k* and manufacturer *i*, and group all $q_{ki}^{R}$s into an *om*-dimensional column vector $Q_{om}^{R}$. $p_{ij}^{N}$ and $p_{ij}^{R}$ are defined as transaction prices of unit new and remanufactured products between manufacturer *i* and retailer *j*, $p_{ki}^{R}$ signifies the price charged by remanufacturer *k* of a unit of remanufactured product. We assume each manufacturer faces a production cost $c_{i}^{N}\left(Q_{m}^{N}\right)$, which might not only depend on its own production but also that of all other manufacturers. For a new product that is manufactured with raw materials, a levy $f_{i}^{N}\left(q_{i}^{N}\right)$ is imposed by the government. $f_{i}^{N}\left(q_{i}^{N}\right)$ is assumed to be monotonically increasing with the production of manufacturer *i*. Let $w_{ij}\left(q_{ij}^{N},q_{ij}^{R}\right)$ denote the transaction cost between manufacturer *i* and retailer *j*, which is associated with transportation, inventory, and transaction, and is assumed to depend, in general, on transaction amounts of both new and remanufactured products. Based on these descriptions, each manufacturer *i* is faced with the following optimization problem:

$$\max\;\pi_{i}=\sum_{j=1}^{n}\left[q_{ij}^{N}p_{ij}^{N}+q_{ij}^{R}p_{ij}^{R}-w_{ij}\left(q_{ij}^{N},q_{ij}^{R}\right)\right]-\sum_{k=1}^{o}q_{ki}^{R}p_{ki}^{R}-c_{i}^{N}\left(Q_{m}^{N}\right)-f_{i}^{N}\left(q_{i}^{N}\right)$$ (1)

subject to

$$q_{i}^{N}\leq \overset{‐}{q}$$ (2)

$$\sum_{j=1}^{n}q_{ij}^{N}\leq q_{i}^{N}$$ (3)

$$\sum_{j=1}^{n}q_{ij}^{R}\leq \sum_{k=1}^{o}q_{ki}^{R}$$ (4)

and the non-negativity constraints: $q_{i}^{N}\geq 0$, $q_{ij}^{N}\geq 0$, $q_{ij}^{R}\geq 0$, and $q_{ki}^{R}\geq 0$.

The first two terms in brackets are the revenues of manufacturer *i* from selling new and remanufactured products to retailers. The other terms show the manufacturer’s transaction cost associated with retailers, payment to remanufacturers, production cost, and levy imposed on the new product, respectively.

Constraint (2) denotes the production capacity constraint. There is a maximum acceptable production output that is technologically available with limited resources for each manufacturer. Inequality (3) ensures that transaction quantity of the new product between the manufacturer and retailers should be less than or equal to the new product output. The transaction quantity of the remanufactured product between the manufacturer–retailer pairs cannot exceed the quantity that manufacturer *i* buys back from all remanufacturers, as in inequality (4).

Furthermore, we assume that all manufactures compete in a non-cooperative fashion. For each manufacturer, we also assume the production and transaction cost functions are convex and continuously differentiable and the utility function is concave. Hence, the optimal conditions for manufacturer *i*, *i* = 1,⋯,*m*, can be expressed as the following variational inequality: determine $\left(Q_{m}^{N*},Q_{mn}^{N*},Q_{mn}^{R*},Q_{om}^{R*},\lambda^{*},\gamma^{*},\mu^{*}\right)\in K_{m}$, satisfying:

$$\begin{array}{l} \sum_{i=1}^{m}\left[\frac{\partial c_{i}^{N}\left(Q_{m}^{N*}\right)}{\partial q_{i}^{N}}+\frac{\partial f_{i}^{N}\left(q_{i}^{N*}\right)}{\partial q_{i}^{N}}+\lambda_{i}^{*}-\gamma_{i}^{*}\right]\left[q_{i}^{N}-q_{i}^{N*}\right]+\\ \sum_{i=1}^{m}\sum_{j=1}^{n}\left[\frac{\partial w_{ij}\left(q_{ij}^{N*},q_{ij}^{R*}\right)}{\partial q_{ij}^{N}}-p_{ij}^{N*}+\gamma_{i}^{*}\right]\left[q_{ij}^{N}-q_{ij}^{N*}\right]+\\ \sum_{i=1}^{m}\sum_{j=1}^{n}\left[\frac{\partial w_{ij}\left(q_{ij}^{N*},q_{ij}^{R*}\right)}{\partial q_{ij}^{R}}-p_{ij}^{R*}+\mu_{i}^{*}\right]\left[q_{ij}^{R}-q_{ij}^{R*}\right]+\sum_{i=1}^{m}\sum_{k=1}^{o}\left[p_{ki}^{R*}-\mu_{i}^{*}\right]\left[q_{ki}^{R}-q_{ki}^{R*}\right]+\\ \sum_{i=1}^{m}\left[{\overset{‐}{q}}_{i}-q_{i}^{N*}\right]\left[\lambda_{i}-\lambda_{i}^{*}\right]+\sum_{i=1}^{m}\left[q_{i}^{N*}-\sum_{j=1}^{n}q_{ij}^{N*}\right]\left[\gamma_{i}-\gamma_{i}^{*}\right]+\sum_{i=1}^{m}\left[\sum_{k=1}^{o}q_{ki}^{R*}-\sum_{j=1}^{n}q_{ij}^{R*}\right]\left[\mu_{i}-\mu_{i}^{*}\right]\geq 0 \end{array}$$ (5)

where $K_{m}\text{=}\left\{\left(Q_{m}^{N},Q_{mn}^{N},Q_{mn}^{R},Q_{om}^{R},\lambda ,\gamma ,\mu \right)|q_{i}^{N}\geq 0,q_{ij}^{N}\geq 0,q_{ij}^{R}\geq 0,q_{ki}^{R}\geq 0,\lambda_{i}\geq 0,\gamma_{i}\geq 0,\right.$ *μ*_*i*_ ≥ 0|∀*i*,*j*,*k*} is a convex set, *λ*, *γ*, and *μ* are the *m*-dimensional Lagrange multipliers associated with constraints (2), (3), and (4), respectively.

#### Proposition 1

For the equilibrium transaction price of new product $p_{ij}^{N*}$, we provide some economic explanations from the abovementioned variational inequality. According to the equivalence between the complementary relation and variational inequality, in the first two terms of Eq (5), when $q_{i}^{N*}>0$ and $q_{ij}^{N*}>0$, we have $\lambda_{i}^{*}=p_{ij}^{N*}-\partial w_{ij}\left(q_{ij}^{N*},q_{ij}^{R*}\right)/\partial q_{ij}^{N}-\partial c_{i}^{N}\left(Q_{m}^{N*}\right)/\partial q_{i}^{N}-\partial f_{i}^{N}\left(q_{i}^{N*}\right)/\partial q_{i}^{N}$. The manufacturer makes the new product only when the transaction price of a unit of product exceeds the summation of the marginal transaction cost, marginal production cost, and unit levy. We also note in the second term of Eq (5) that $p_{ij}^{N*}$ increases when the marginal transaction cost between manufacturer *i* and retailer *j* increases.

### The behaviour of retailers and their optimality conditions

Retailers in this model undertake transactions with manufacturers in order to cope with fuzzy demand while still seeking to maximize their profits. Let $q_{j}^{N}$ and $q_{j}^{R}$ denote the respective order volumes of retailer *j* for new and remanufactured products from manufacturers, $q_{j}^{N}=\sum_{i=1}^{m}q_{ij}^{N}$ and $q_{j}^{R}=\sum_{i=1}^{m}q_{ij}^{R}$. We group all order volumes of the new and remanufactured products into $Q_{n}^{N}$ and $Q_{n}^{R}$. Each retailer is faced with a transaction cost $w_{j}\left(Q_{n}^{N},Q_{n}^{R}\right)$ for marketing, which may include, as appropriate, any selling expenses and management fee for business with consumers in the demand market and can generally depend on the order volumes of all retailers of both products. Considering product heterogeneity and changeable consumer preferences for differentiated products, the demand of products is uncertain. We assume $\tilde{d_{j}^{N}}$ as the fuzzy demand for the new product, and $\tilde{d_{j}^{R}}$ as that for the remanufactured one. $p_{j}^{N}$ and $p_{j}^{R}$ are respective sales prices. The optimization problem of retailer *j* is expressed as

$$\max\;\pi_{j}=p_{j}^{N}\min\left(\tilde{d_{j}^{N}},q_{j}^{N}\right)+p_{j}^{R}\min\left(\tilde{d_{j}^{R}},q_{j}^{R}\right)-\sum_{i=1}^{m}\left[q_{ij}^{N}p_{ij}^{N}+q_{ij}^{R}p_{ij}^{R}\right]-w_{j}\left(Q_{n}^{N},Q_{n}^{R}\right)$$ (6)

subject to

$$q_{j}^{N}=\sum_{i=1}^{m}q_{ij}^{N}$$ (7)

$$q_{j}^{R}=\sum_{i=1}^{m}q_{ij}^{R}$$ (8)

The first two terms in Eq (6) are identified as the expected revenues of the retailer; the latter two terms are the pay-out to the manufacturers and the transaction cost with the market. Constraints (7) and (8) show the flow conservation between manufacturers and retailer *j*.

According to Eq (6), the expected profits of retailer *j* can be deduced after algebraic simplification as

$$\begin{array}{lll} \text{max}\;E\left(\pi_{j}\right) & = & p_{j}^{N}q_{j}^{N}-p_{j}^{N}\overset{q_{j}^{N}}{\underset{\underset{‐}{d_{j}^{N}}}{\int }}\left(q_{j}^{N}-x\right)d\sigma_{j}^{N}\left(x\right)+p_{j}^{R}q_{j}^{R}-p_{j}^{R}\overset{q_{j}^{R}}{\underset{\underset{‐}{d_{j}^{R}}}{\int }}\left(q_{j}^{R}-x\right)d\sigma_{j}^{R}\left(x\right)\\ & & -\overset{m}{\underset{i=1}{\sum }}\left[q_{ij}^{N}p_{ij}^{N}+q_{ij}^{R}p_{ij}^{R}\right]-w_{j}\left(Q_{n}^{N},Q_{n}^{R}\right) \end{array}$$ (9)

where $\underset{‐}{d_{j}^{N}}$ and $\underset{‐}{d_{j}^{R}}$ represent the lower limits of market demand for the new and remanufactured products, respectively. $\sigma_{j}^{N}\left(x\right)$ and $\sigma_{j}^{R}\left(x\right)$ are defined as the credibility distribution functions. The proof of Eq (9) and its qualitative properties are provided in S2 Appendix.

We assume that the transaction cost is convex and continuously differentiable and the retailers compete non-cooperatively. Then the optimality conditions for retailer *j* can be formulated as the following variational inequality: determine $\left(Q_{mn}^{N*},Q_{mn}^{R*},Q_{n}^{N*},Q_{n}^{R*},\eta^{*},\varepsilon^{*}\right)\in K_{n}$ such that

$$\begin{array}{l} \sum_{j=1}^{n}\sum_{i=1}^{m}\left[p_{ij}^{N*}+\frac{\partial w_{j}\left(Q_{n}^{N*},Q_{n}^{R*}\right)}{\partial q_{ij}^{N}}-\eta_{j}^{*}\right]\left[q_{ij}^{N}-q_{ij}^{N*}\right]+\sum_{j=1}^{n}\left[p_{j}^{N*}\sigma_{j}^{N}\left(x\right)-p_{j}^{N*}+\eta_{j}^{*}\right]\left[q_{j}^{N}-q_{j}^{N*}\right]+\\ \sum_{j=1}^{n}\sum_{i=1}^{m}\left[p_{ij}^{R*}+\frac{\partial w_{j}\left(Q_{n}^{N*},Q_{n}^{R*}\right)}{\partial q_{ij}^{R}}-\varepsilon_{j}^{*}\right]\left[q_{ij}^{R}-q_{ij}^{R*}\right]+\sum_{j=1}^{n}\left[p_{j}^{R*}\sigma_{j}^{R}\left(x\right)-p_{j}^{R*}+\varepsilon_{j}^{*}\right]\left[q_{j}^{R}-q_{j}^{R*}\right]+\\ \sum_{j=1}^{n}\left[\sum_{i=1}^{m}q_{ij}^{N*}-q_{j}^{N*}\right]\left[\eta_{j}-\eta_{j}^{*}\right]+\sum_{j=1}^{n}\left[\sum_{i=1}^{m}q_{ij}^{R*}-q_{j}^{R*}\right]\left[\varepsilon_{j}-\varepsilon_{j}^{*}\right]\geq 0 \end{array}$$ (10)

where $K_{n}\text{=}\left\{\left(Q_{mn}^{N},Q_{mn}^{R},Q_{n}^{N},Q_{n}^{R},\eta ,\varepsilon \right)|q_{ij}^{N}\geq 0,q_{ij}^{R}\geq 0,q_{j}^{N}\geq 0,q_{j}^{R}\geq 0,\eta_{j}\geq 0,\varepsilon_{j}\geq 0|\forall i,j\right\}$ is a convex set. *η* and *ε* are *n*-dimensional Lagrange multipliers associated with constraints (7) and (8).

#### Proposition 2

For the equilibrium sales prices of new and remanufactured products $p_{j}^{N*}$, $p_{j}^{R*}$, when $q_{ij}^{N*}>0$ and $q_{j}^{N*}>0$ can be obtained from the first two terms in Eq (10), we have $p_{j}^{N*}=\left[p_{ij}^{N*}+\partial w_{j}\left(Q_{n}^{N*},Q_{n}^{R*}\right)/\partial q_{ij}^{N}\right]/\left[1-\sigma_{j}^{N}\left(x\right)\right]$, which reflects that the sales price of a unit of new product increases with greater transaction price, competitive transaction cost, and market demand uncertainty. The same economic explanation holds for the remanufactured product after algebraic computation of the third and fourth terms in Eq (10), to obtain $p_{j}^{R*}=\left[p_{ij}^{R*}+\partial w_{j}\left(Q_{n}^{N*},Q_{n}^{R*}\right)/\partial q_{ij}^{R}\right]/$$\left[1-\sigma_{j}^{R}\left(x\right)\right]$.

### The behaviour of remanufacturers and their optimality conditions

Remanufacturer *k* collects EOL product from the demand market at a unit price of $p_{k}^{E}$, then remanufactures and sells the remanufactured product charged at $p_{ki}^{R}$ to the manufacturers. Each remanufacturer competes to find the optimal remanufacturing output of the remanufactured product $q_{k}^{R}$, the transaction quantity between the manufacturers $q_{ki}^{R}$, and EOL product recycling quantity from the demand market $q_{k}^{E}$. We group these variables into vectors $Q_{o}^{R}$, $Q_{om}^{R}$, and $Q_{o}^{E}$, respectively. Remanufacturer *k* incurs a transaction cost between each manufacturer $w_{ki}\left(q_{ki}^{R}\right)$ and that between the demand market $w_{k}\left(q_{k}^{E}\right)$, a competitive remanufacturing cost $c_{k}^{R}\left(Q_{o}^{R}\right)$ associated with all remanufacturers. The government provides subsidy $s_{k}^{R}\left(q_{k}^{R}\right)$ for the remanufactured product to encourage remanufacturing operations. The profit maximization for remanufacturer *k* can be expressed as

$$\max\;\pi_{k}=\sum_{i=1}^{m}\left[q_{ki}^{R}p_{ki}^{R}-w_{ki}\left(q_{ki}^{R}\right)\right]-w_{k}\left(q_{k}^{E}\right)-q_{k}^{E}p_{k}^{E}-c_{k}^{R}\left(Q_{o}^{R}\right)+s_{k}^{R}\left(q_{k}^{R}\right)$$ (11)

subject to

$$\sum_{i=1}^{m}q_{ki}^{R}\leq q_{k}^{R}$$ (12)

$$q_{k}^{R}\leq q_{k}^{E}$$ (13)

The first term in the bracket of objective function (11) is the revenue of remanufacturer *k* from selling remanufactured products to manufacturers. Other terms include the transaction cost of the remanufactured and EOL products, the purchase of the EOL product, the competitive remanufacturing cost, and the subsidy provided by government. Constraints (12) and (13) ensure that the remanufacturing output of remanufacturer *k* is larger than the transaction quantity between the remanufacturer and all manufacturers, and should not exceed the EOL product recycling amount from the market.

We assume that $w_{ki}\left(q_{ki}^{R}\right)$, $w_{k}\left(q_{k}^{E}\right)$, and $c_{k}^{R}\left(Q_{o}^{R}\right)$ are convex and continuously differentiable. Hence, the optimal conditions for remanufacturer *k* can be expressed as the following variational inequality: determine $\left(Q_{o}^{R*},Q_{om}^{R*},Q_{o}^{E*},\beta^{*},\rho^{*}\right)\in K_{o}$, satisfying:

$$\begin{array}{l} \sum_{k=1}^{o}\left[\frac{\partial c_{k}^{R}\left(Q_{o}^{R*}\right)}{\partial q_{k}^{R}}-\frac{\partial s_{k}^{R}\left(q_{k}^{R*}\right)}{\partial q_{k}^{R}}-\beta_{k}^{*}+\rho_{k}^{*}\right]\left[q_{k}^{R}-q_{k}^{R*}\right]+\\ \sum_{k=1}^{o}\sum_{i=1}^{m}\left[\frac{\partial w_{ki}\left(q_{ki}^{R*}\right)}{\partial q_{ki}^{R}}-p_{ki}^{R*}+\beta_{k}^{*}\right]\left[q_{ki}^{R}-q_{ki}^{R*}\right]+\\ \sum_{k=1}^{o}\left[p_{k}^{E*}+\frac{\partial w_{k}\left(q_{k}^{E*}\right)}{\partial q_{k}^{E}}-\rho_{k}^{*}\right]\left[q_{k}^{E}-q_{k}^{E*}\right]+\\ \sum_{k=1}^{o}\left[q_{k}^{R*}-\sum_{i=1}^{m}q_{ki}^{R*}\right]\left[\beta_{k}-\beta_{k}^{*}\right]+\sum_{k=1}^{o}\left[q_{k}^{E*}-q_{k}^{R*}\right]\left[\rho_{k}-\rho_{k}^{*}\right]\geq 0 \end{array}$$ (14)

where $K_{o}\text{=}\left\{\left(Q_{o}^{R},Q_{om}^{R},Q_{o}^{E},\beta ,\rho \right)|q_{k}^{R}\geq 0,q_{ki}^{R}\geq 0,q_{k}^{E}\geq 0,\beta_{k}\geq 0,\rho_{k}\geq 0|\forall k,i\right\}$ is a convex set. *β* and *ρ* are *o*-dimensional Lagrange multipliers associated with constraints (12) and (13), respectively.

#### Proposition 3

1. For the relations of remanufacturing cost $c_{k}^{R*}$ and government subsidy $s_{k}^{R*}$, the first term in Eq (14) indicates that when the marginal remanufacturing cost increases, the unit subsidy also increases. In other words, the increase of government subsidy to some degree compensates for the rising cost and enhances remanufacturing implementation.
2. For the equilibrium transaction price of remanufactured product $p_{ki}^{R*}$, when $q_{ki}^{R*}>0$ is satisfied from the second term in Eq (14) , the transaction price $p_{ki}^{R*}$ equals the summation of the marginal transaction cost of remanufacturer–manufacturer pairs $\partial w_{ki}^{*}/\partial q_{ki}^{R*}$ and the Lagrange multiplier $\beta_{k}^{*}$.

### The behaviour of consumers in the demand market and their optimality conditions

In the forward RSC network, consumers purchase the products under a price charged by the retailers $p_{j}^{N}$ and $p_{j}^{R}$ for a unit of new and remanufactured products, respectively. The market demand is uncertain, functions of fuzzy demand $\tilde{d_{j}^{N}}$ and $\tilde{d_{j}^{R}}$ may depend, in general, on the entire demand price pattern and different price competitiveness, as well as on consumer preferences for differentiated products, since consumers within the demand market respond not only to products’ prices but also to their origins. Let *τ*^*N*^ and *τ*^*R*^ denote the price competitiveness of new and remanufactured products, respectively, and let *δ* denote consumer preference for the new product, so that (1 ‒ *δ*) represents preference for the remanufactured product, *δ* ∈ [0,1]. The significant value of (1 ‒ *δ*) reflects enhanced environmental preference of the consumers. Thus, $d_{j}^{N}$ and $\tilde{d_{j}^{R}}$ can be expressed by $\tilde{d_{j}^{N}}=\tilde{d_{j}^{N}}\left(p_{j}^{N},p_{j}^{R},\tau^{N},\delta \right)$, $\tilde{d_{j}^{R}}=\tilde{d_{j}^{R}}\left(p_{j}^{N},p_{j}^{R},\tau^{R},\delta \right)$.

The equilibrium conditions associated with the transactions that take place between the retailers and consumers are stochastic economic equilibrium conditions [47–49], which mathematically take the following form for any retailer *j*:

$$E\left[\tilde{d_{j}^{N}}\right]=\left\{\begin{array}{l} =q_{j}^{N*},p_{j}^{N*}>0\\ \leq q_{j}^{N*},p_{j}^{N*}=0 \end{array}\right.$$ (15)

$$E\left[\tilde{d_{j}^{R*}}\right]=\left\{\begin{array}{l} =q_{j}^{R*},p_{j}^{R*}>0\\ \leq q_{j}^{R*},p_{j}^{R*}=0 \end{array}\right.$$ (16)

According to Eqs (15) and (16), on the one hand, if the sales price of a product in the demand market is zero, then the product supply can exceed the demand. On the other hand, if the sales price that retailer *j* charges and that consumers are willing to pay is positive, then the quantities purchased by the retailer from the manufacturers in the aggregate are equal to the demand.

In the reverse supply chain, consumers return the EOL product to the remanufacturers at some price. Their behaviour can be characterized by Eq (17).

$$\alpha \left(Q_{o}^{E}\right)=\left\{\begin{array}{l} =p_{k}^{E*},q_{k}^{E*}>0\\ \geq p_{k}^{E*},q_{k}^{E*}=0 \end{array}\right.$$ (17)

subject to

$$\sum_{k=1}^{o}q_{k}^{E}\leq \sum_{j=1}^{n}\left[\min\left(\tilde{d_{j}^{N}},q_{j}^{N}\right)\right]$$ (18)

where *α* refers to the disutility that consumers face for returning EOL products. We assume that *α* is an increasing function of the total recycle volume $Q_{o}^{E}$, which signifies that the more consumers return the EOL products, the more inconvenience they will encounter. $p_{k}^{E}$ represents the price of EOL products that remanufacturer *k* pays to collect from the demand market. Eq (17) suggests that if a manufacturer opts out in remanufacturing (outsourced remanufacturing in the current model), then consumer compensation for returning the EOL product is smaller than the disutility. On the contrary, if a manufacturer decides to outsource remanufacturing to a 3PR, then the disutility of consumers could be compensated. Thus, there is a positive quantity of the EOL product transacted from the demand market to remanufacturer *k* in equilibrium, if the disutility of consumers equals the recycling price of a unit of EOL product. Constraint (18) states that the amount of the recycled EOL products must not exceed the new products sold in the market.

By combining consumer behaviour in both the forward and reverse chains, the equilibrium conditions of the demand market can be formulated as the following variational inequality: determine $\left(Q_{o}^{E*},Q_{n}^{N*},Q_{n}^{R*},P_{n}^{N*},P_{n}^{N*},\xi^{*}\right)\in K_{c}$, satisfying:

$$\begin{array}{l} \sum_{j=1}^{n}\left[q_{j}^{N*}-E\left[\tilde{d_{j}^{N}}\right]\right]\left[p_{j}^{N}-p_{j}^{N*}\right]+\\ \sum_{j=1}^{n}\left[q_{j}^{R*}-E\left[\tilde{d_{j}^{R}}\right]\right]\left[p_{j}^{R}-p_{j}^{R*}\right]+\sum_{k=1}^{o}\left[\alpha \left(Q_{o}^{E*}\right)-p_{k}^{E*}+\xi^{*}\right]\left[q_{k}^{E}-q_{k}^{E*}\right]+\\ \left[\sum_{j=1}^{n}\left[q_{j}^{N*}-\int_{\underset{‐}{d_{j}^{N}}}^{v_{j}^{N}}\left(q_{j}^{N}-x\right)d\sigma_{j}^{N}\left(x\right)\right]-\sum_{k=1}^{o}q_{k}^{E}\right]\left[\xi -\xi^{*}\right]\geq 0 \end{array}$$ (19)

where $K_{c}\text{=}\left\{\left(Q_{o}^{E},Q_{n}^{N},Q_{n}^{R},P_{n}^{N},P_{n}^{R},\xi \right)|q_{k}^{E}\geq 0,q_{j}^{N}\geq 0,q_{j}^{R}\geq 0,p_{j}^{N}\geq 0,p_{j}^{R}\geq 0,\xi \geq 0|\forall j,k\right\}$ is a convex set, and *ξ* is the Lagrange multiplier associated with constraint (18). In the third term of Eq (19), when $q_{k}^{E*}>0$, the optimal EOL product collection price $p_{k}^{E*}$ equals the summation of consumer disutility *α* and the Lagrange multiplier ξ^*^.

### The equilibrium conditions of the RSC network

In equilibrium, the aggregation of the optimally conditions for all manufacturers, retailers, remanufacturers, and the demand market, as expected by conditions (5), (10), (14), and (19), respectively, must be satisfied. Hence, the transaction quantity that the manufacturers sell to the retailers must equal the quantity that the retailers accept from manufacturers. The same holds for the transaction quantity between the manufacturers and remanufacturers, and that between the demand market and remanufacturers. We denote the network equilibrium explicitly as: In the equilibrium state of the RSC network, the product flows between distinct tiers of decision-makers coincide and prices satisfy the summation of the optimality conditions of Eqs (5), (10), (14), and (19).

**Theorem 1** (*variational inequality formulation*). The equilibrium conditions governing the RSC network model are equivalent to the solution of the variational inequality problem expressed by: determine $\left(Q_{m}^{N*},Q_{o}^{R*},Q_{mn}^{N*},Q_{mn}^{R*},Q_{om}^{R*},Q_{o}^{E*},Q_{n}^{N*},Q_{n}^{R*},\right.$ $\left.P_{n}^{N*},P_{n}^{R*},\lambda^{*},\gamma^{*},\mu^{*},\eta^{*},\varepsilon^{*},\beta^{*},\rho^{*},\xi^{*}\right)\in K$, satisfying:

$$\begin{array}{l} \sum_{i=1}^{m}\left[\frac{\partial c_{i}^{N}\left(Q_{m}^{N*}\right)}{\partial q_{i}^{N}}+\frac{\partial f_{i}^{N}\left(q_{i}^{N*}\right)}{\partial q_{i}^{N}}+\lambda_{i}^{*}-\gamma_{i}^{*}\right]\left[q_{i}^{N}-q_{i}^{N*}\right]+\\ \sum_{k=1}^{o}\left[\frac{\partial c_{k}^{R}\left(Q_{o}^{R*}\right)}{\partial q_{k}^{R}}-\frac{\partial s_{k}^{R}\left(q_{k}^{R*}\right)}{\partial q_{k}^{R}}-\beta_{k}^{*}+\rho_{k}^{*}\right]\left[q_{k}^{R}-q_{k}^{R*}\right]+\\ \sum_{i=1}^{m}\sum_{j=1}^{n}\left[\frac{\partial w_{ij}\left(q_{ij}^{N*}\right)}{\partial q_{ij}^{N}}+\frac{\partial w_{j}\left(q_{ij}^{N*}\right)}{\partial q_{ij}^{N}}+\gamma_{i}^{*}-\eta_{j}^{*}\right]\left[q_{ij}^{N}-q_{ij}^{N*}\right]+\\ \sum_{i=1}^{m}\sum_{j=1}^{n}\left[\frac{\partial w_{ij}\left(q_{ij}^{R*}\right)}{\partial q_{ij}^{R}}+\frac{\partial w_{j}\left(q_{ij}^{R*}\right)}{\partial q_{ij}^{R}}+\mu_{i}^{*}-\varepsilon_{j}^{*}\right]\left[q_{ij}^{R}-q_{ij}^{R*}\right]+\\ \sum_{k=1}^{o}\sum_{i=1}^{m}\left[\frac{\partial w_{ki}\left(q_{ki}^{R*}\right)}{\partial q_{ki}^{R}}+\beta_{k}^{*}-\mu_{i}^{*}\right]\left[q_{ki}^{R}-q_{ki}^{R*}\right]+\sum_{k=1}^{o}\left[\alpha +\frac{\partial w_{k}\left(q_{k}^{E*}\right)}{\partial q_{k}^{E}}-\rho_{k}^{*}+\xi^{*}\right]\left[q_{k}^{E}-q_{k}^{E*}\right]+\\ \sum_{j=1}^{n}\left[p_{j}^{N*}\sigma_{j}^{N}\left(x\right)-p_{j}^{N*}+\eta_{j}^{*}\right]\left[q_{j}^{N}-q_{j}^{N*}\right]+\sum_{j=1}^{n}\left[p_{j}^{R*}\sigma_{j}^{R}\left(x\right)-p_{j}^{R*}+\varepsilon_{j}^{*}\right]\left[q_{j}^{R}-q_{j}^{R*}\right]+\\ \sum_{j=1}^{n}\left[q_{j}^{N*}-E\left[\tilde{d_{j}^{N}}\right]\right]\left[p_{j}^{N}-p_{j}^{N*}\right]+\sum_{j=1}^{n}\left[q_{j}^{R*}-E\left[\tilde{d_{j}^{R}}\right]\right]\left[p_{j}^{R}-p_{j}^{R*}\right]+\\ \sum_{i=1}^{m}\left[{\overset{‐}{q}}_{i}-q_{i}^{N*}\right]\left[\lambda_{i}-\lambda_{i}^{*}\right]+\sum_{i=1}^{m}\left[q_{i}^{N*}-\sum_{j=1}^{n}q_{ij}^{N*}\right]\left[\gamma_{i}-\gamma_{i}^{*}\right]+\sum_{i=1}^{m}\left[\sum_{k=1}^{o}q_{ki}^{R*}-\sum_{j=1}^{n}q_{ij}^{R*}\right]\left[\mu_{i}-\mu_{i}^{*}\right]+\\ \sum_{j=1}^{n}\left[\sum_{i=1}^{m}q_{ij}^{N*}-q_{j}^{N*}\right]\left[\eta_{j}-\eta_{j}^{*}\right]+\sum_{j=1}^{n}\left[\sum_{i=1}^{m}q_{ij}^{R*}-q_{j}^{R*}\right]\left[\varepsilon_{j}-\varepsilon_{j}^{*}\right]+\\ \sum_{k=1}^{o}\left[q_{k}^{R*}-\sum_{i=1}^{m}q_{ki}^{R*}\right]\left[\beta_{k}-\beta_{k}^{*}\right]+\sum_{k=1}^{o}\left[q_{k}^{E*}-q_{k}^{R*}\right]\left[\rho_{k}-\rho_{k}^{*}\right]+\\ \left[\sum_{j=1}^{n}\left[q_{j}^{N*}-\int_{\underset{‐}{d_{j}^{N}}}^{v_{j}^{N}}\left(q_{j}^{N}-x\right)d\sigma_{j}^{N}\left(x\right)\right]-\sum_{k=1}^{o}q_{k}^{E}\right]\left[\xi -\xi^{*}\right]\geq 0 \end{array}$$ (20)

$\forall \left(Q_{m}^{N},Q_{o}^{R},Q_{mn}^{N},Q_{mn}^{R},Q_{om}^{R},Q_{o}^{E},Q_{n}^{N},Q_{n}^{R},P_{n}^{N},P_{n}^{R},\lambda ,\gamma ,\mu ,\eta ,\varepsilon ,\beta ,\rho ,\xi \right)\in K$ where *K* = *K*_*m*_ × *K*_*n*_ × *K*_*o*_ × *K*_*c*_.

The proof of Theorem 1, the closed-form solutions of the RSC network prices, and the qualitative studies are provided in S3-S5 Appendices.

### Numerical examples

In this section, a practical case of Hangzhou Good Friend Precision Machinery Co., Ltd. (Good Friend Precision Machinery) is cited primarily, then results of questionnaire survey and field research about consumer preference for remanufactured products in Yangtze River Delta of China are introduced. Based on real data and the proposed RSC network equilibrium model, we attempt to investigate the influences of consumer preference, production capacity constraint, price competitiveness, market demand fluctuation and government financial measures on RSC network strategies.

### Real case and questionnaire survey

Good Friend Precision Machinery is a specialized enterprise in the manufacture and export of numerical control machines. It has also been engaged in remanufacturing waste machine tools for years. It collects and remanufactures EOL products when a client enterprise sends the order for remanufacturing. Thus, Good Friend Precision Machinery is referred to as the remanufacturer in the model.

Considering the role of consumers in affecting remanufacturing decisions, we conduct questionnaire survey and field research on public awareness of remanufactured product in Yangtze River Delta, which is one of the largest delta areas in China with a coverage area of 210,000 km^2^ and a population of 160 million. We distributed 700 questionnaires and received 611 valid ones (The questionnaire, field research, and survey findings are provided in S6 Appendix). The survey results show that consumer recognition of remanufactured products (the proportion of respondents who have heard of remanufactured products) is 53.36%, and purchase experience of remanufactured products (the proportion of respondents who have purchased remanufactured products) is 30.44%.

The regulation of “*Swap the old for remanufacturing*” executed by Chinese government stipulates that subsidies should be offered for remanufactured products regarding automobile engines, transmission cases, and large machine tools, among others. The government provides 10% of the replacement value for the remanufactured products possessed with domestic certifications and qualified for sale, while 2000 CNY (~$310) at most would be allocated for a unit of remanufactured product. In the case that Good Friend Precision Machinery acts as the remanufacturer, unit subsidy of 1750 CNY (~$271) is offered. The monetary data is scaled down in proportion, 1000 times specifically, to make following analyses reasonable and convenient, thus *s* = 1.75 as the subsidy provided for unit remanufactured product is set accordingly.

### Initial parameters and functions setting

We take advantage of the data accumulated from Good Friend Precision Machinery and questionnaire survey, and refer to the work of Zhou et al. [50], Zhang et al. [51], Zhou et al. [52], to define initial parameters and functions. In any functions related to production and transaction costs, the linear terms reflect the unit costs and the quadratic terms indicate that the marginal cost increases as the quantity approaches the maximum capacity. The RSC network consists of two manufacturers *m* = 2, two retailers *n* = 2, two remanufacturers *o* = 2, and one typical demand market. Elementary functions are set as follows (*i* = 1,2, *j* = 1,2, *k* = 1,2), the manufacturers’ production cost functions are defined as $c_{i}^{N}\left(Q_{m}^{N}\right)=2.5{\left(q_{i}^{N}\right)}^{2}+q_{i}^{N}q_{2-i}^{N}+2q_{i}^{N}$, the transaction costs between manufacturers and retailers are $w_{ij}\left(q_{ij}^{N},q_{ij}^{R}\right)=0.25{\left(q_{ij}^{N}+q_{ij}^{R}\right)}^{2}+2.5\left(q_{ij}^{N}+q_{ij}^{R}\right)$, the levy functions imposed on the manufacturers are $f_{i}^{N}\left(q_{i}^{N}\right)=1.25q_{i}^{N}$, the transaction cost functions of retailers are $w_{j}\left(Q_{n}^{N},Q_{n}^{R}\right)=0.5\left[{\left(q_{ij}^{N}+q_{\left(3-i\right)j}^{N}\right)}^{2}+\right.\left.{\left(q_{ij}^{r}+q_{\left(3-i\right)j}^{R}\right)}^{2}\right]+\left(q_{ij}^{N}+q_{\left(3-i\right)j}^{N}\right)\left(q_{ij}^{R}+q_{\left(3-i\right)j}^{R}\right)$ $+\left[\left(q_{ij}^{N}+q_{\left(3-i\right)j}^{N}\right)+\left(q_{ij}^{R}+q_{\left(3-i\right)j}^{R}\right)\right]$, the remanufacturing cost functions of remanufacturers are $c_{k}^{R}\left(Q_{o}^{R}\right)=2{\left(q_{k}^{R}\right)}^{2}+q_{k}^{R}q_{2-k}^{R}+2q_{k}^{R}$, the subsidies provided by the government for remanufacturers are $s_{k}^{R}\left(q_{k}^{R}\right)=1.75q_{k}^{R}$, the transaction cost functions between remanufacturers and manufacturers are $w_{ki}\left(q_{ki}^{R}\right)=0.5{\left(q_{ki}^{R}\right)}^{2}+0.5q_{ki}^{R}$, the transaction cost functions between remanufacturers and the demand market are $w_{k}\left(q_{k}^{E}\right)=0.5q_{k}^{E}+3$. The disutility functions of consumers in the demand market are defined as $\alpha \left(Q_{o}^{E}\right)=0.5\left[q_{k}^{E}+q_{\left(3-k\right)}^{E}\right]+2$.

The new and remanufactured products sold in the demand market are distinct but substitutable. Consumer preference for the new product δ and that for the remanufactured product (1 ‒ *δ*) should not exceed 1. A consumer with a high (low) value of *δ* indicates that he/she values the remanufactured product less (more). $\tilde{d_{j}^{N}}\left(p_{j}^{N},p_{j}^{R},\tau^{N},\delta \right)$ and $\tilde{d_{j}^{R}}\left(p_{j}^{N},p_{j}^{R},\tau^{R},\delta \right)$ are deemed as triangle distributions $\left[b_{j}^{N}/p_{j}^{N}-\underset{‐}{\Delta },b_{j}^{N}/p_{j}^{N},b_{j}^{N}/p_{j}^{N}+\overset{‐}{\Delta }\right]$ and $\left[b_{j}^{R}/p_{j}^{R}-\underset{‐}{\Delta },b_{j}^{R}/p_{j}^{R},b_{j}^{R}/p_{j}^{R}+\overset{‐}{\Delta }\right]$, respectively, where $b_{j}^{N}=\left(1+\delta \right)\left\{\delta \cdot M+\tau^{N}\left[p_{j}^{R}+p_{\left(3-j\right)}^{R}\right]\right\}$ and $b_{j}^{R}=\left(1-\delta \right)\cdot M+\tau^{R}\left[p_{j}^{N}+p_{\left(3-j\right)}^{N}\right]$, *τ*^*N*^ = 0.3 and *τ*^*R*^ = 0.1 are initial values of the price competitiveness of the new and remanufactured products, respectively. $\underset{‐}{\Delta }=\overset{‐}{\Delta }=10$ is defined as the upper and inferior limits of market demand fluctuation, and *M* = 400 represents the market demand space of both new and remanufactured products. $b_{j}^{N}$ and $b_{j}^{R}$ in the demand function include two parts: demand affected by consumer preference and demand fluctuation caused by price competitiveness between the new and remanufactured products [52]. Such an allocation of $b_{j}^{N}$ and $b_{j}^{R}$ ensures that market demand would decrease with increased sales prices of both products, demand of the remanufactured product increases with elevated consumer environmental awareness and increased sales price of the new product. Consumers are willing to buy and pay more for the remanufactured product as their environmental awareness and preference for the remanufactured product improve, which is in line with the actual situation [10, 25].

We present several numerical examples based on the above initial parameters and functions, and adopt the logarithmic-quadratic proximal prediction-correction (LQP-PC) method for variational inequality to obtain equilibrium solutions [53, 54]. The convergence tolerance is 10^−8^, so that the algorithm is deemed to have converged when the absolute value of the difference between each successive production output, remanufacturing output, product transaction, transaction price, and sales price is less than or equal to 10^−8^. We initialize the algorithm by setting all decision variables at 0, and the Lagrange multipliers at 1. The algorithm is presented in S7 Appendix.

### Numerical analysis

In this subsection, we provide several numerical examples to elaborate influences of consumer preference, production capacity constraint, price competitiveness, market demand fluctuation and government fiscal regulation, on remanufacturing related strategies.

#### Example I: Influences of consumer preference on RSC network strategies

In this example, consumer preference for the new product *δ* is assumed to vary from [0,1], thus impacts of consumer preference variance on RSC network strategies could be captured.

**Fig 2:**
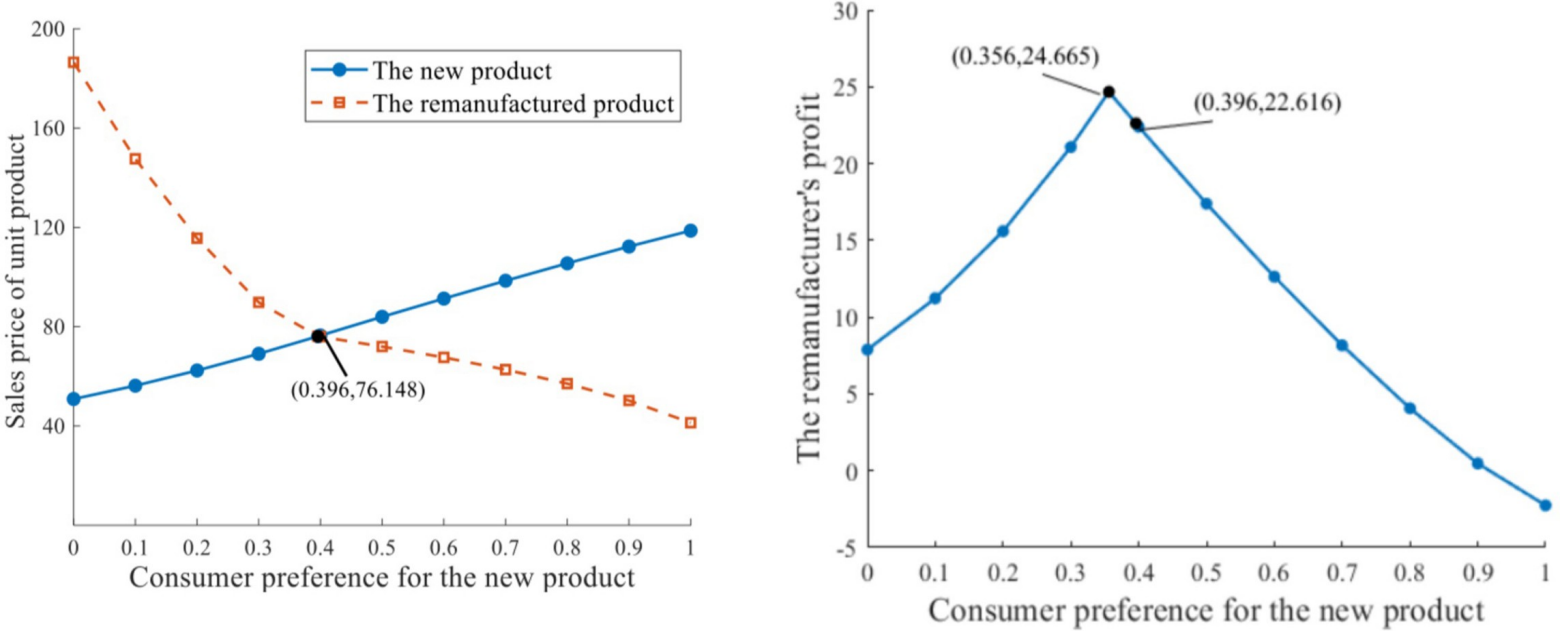
Influence of consumer preference with unlimited production capacity. **a.** Sales price of unit product. **b.** Profit of the remanufacturer.

*ScenarioI(1)*. Under unlimited production capacity (i.e. $\overset{‐}{q}=+\infty$), the influences of various consumer preferences, *δ*∈[0,1], are depicted in Fig 2.

The sales price of a unit of remanufactured product at equilibrium $p_{j}^{R*}$ increases with the decrease of consumer preference for the new product *δ*. When *δ* is within a certain range, *δ*∈[0,0.396] in the case, $p_{j}^{R*}>p_{j}^{N*}$; otherwise, pricing for the new product should be higher from *δ* = 0.396 onwards. When consumers prefer the remanufactured product at a level of 1 ‒ *δ* = 0.604, $p_{j}^{N*}=p_{j}^{R*}=76.148$.

The profit of the remanufacturer at equilibrium $\pi_{k}^{*}$ first improves and then reduces with an increase of *δ*. If the remanufactured product has not gained much recognition and the demand market values the new product highly, remanufacturing appears to be unprofitable, with the remanufacturer’s profit evolving to negative values as *δ* approaches 1. When *δ* reduces to 0.396 or less, consumers value the remanufactured product more than the new one, such that pricing for the remanufactured product should be higher from the changeover point onward. There should be more recognition and acceptance of the remanufactured product, at a level of 1 ‒ *δ* = 0.644, such that the maximum profit of the remanufacturer at equilibrium can be 24.665. Nonetheless, if consumers have an even higher preference for the remanufactured product, *δ*∈ = (0,0.356] in Fig 2(B), being proactive in remanufacturing is less attractive.

**Fig 3:**
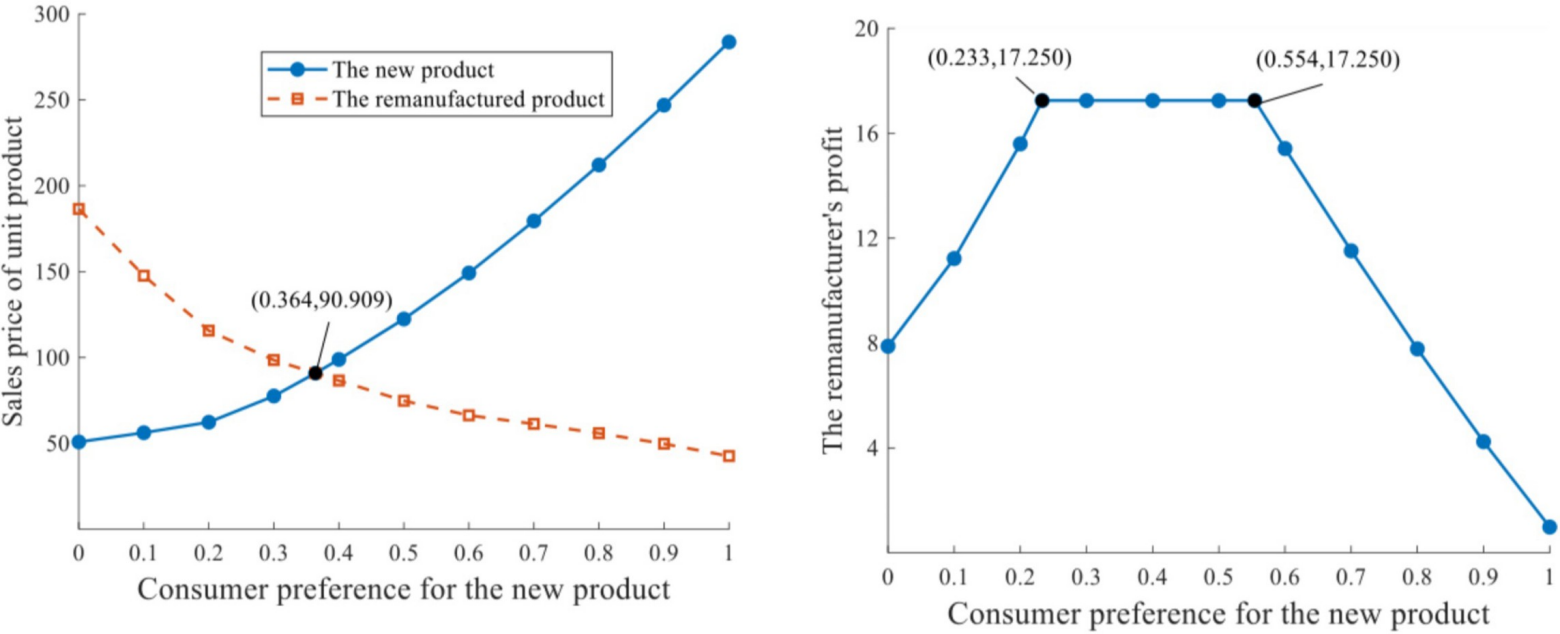
Influence of consumer preference with limited production capacity of 3. **a.** Sales price of unit product. **b.** Profits of the remanufacturer.

*Scenario I(2)*. This scenario is the same as Scenario I(1), except that production capacity constraint, $\overset{‐}{q}=3$ is allocated owing to limited resources and technology available for each manufacturer. The variation trends of sales prices and remanufacturer’s profit are depicted in Fig 3.

The variation trends of sales prices at equilibrium presented in Fig 3(A) are similar to those in Fig 2(B) while the prices are more responsive to the change of consumer preference. The critical value of *δ* declines slightly: *δ* = 0.364 makes $p_{j}^{N*}=p_{j}^{R*}=90.909$. Similar to Scenario I(1), the remanufacturer’s profit is concavely related to consumer preference for the new product. When consumers in the demand market value the remanufactured product at a level of [0.446, 0.767] (δ∈[0.233,0.554)in Fig 3(B)), the maximum $\pi_{k}^{*}=17.250$ can be achieved, which is devalued relative to the maximum profit in Fig 2(B). By this moment, production output of the new product $q_{i}^{N*}=3$ and the remanufacturing output $q_{k}^{R*}=3$ can be obtained, from which the actual remanufacturing rate $\theta^{*}=q_{k}^{R*}/\left(q_{i}^{N*}+q_{k}^{R*}\right)=0.5$ is deduced. When *δ* opts out a certain range (i.e. *δ*∈[0,0.233)and *δ*∈(0.554,1], which indicates either excessive or deficient consumer preference for the remanufactured product), both $\pi_{k}^{*}$ and θ^*^ reduce.

#### Example II: Influences of production capacity constraint on RSC network strategies

We now investigate remanufacturing-related undertakings and performances under distinct production capacity constraints, unlimited production ($\overset{‐}{q}=+\infty$), limited production capacity of 4 ($\overset{‐}{q}=4$), and limited production capacity of 3 ($\overset{‐}{q}=3$) in sequence. These contradictory situations generate comparisons of the equilibrium solutions as well as the effects of consumer preference on devising strategy.

**Fig 4:**
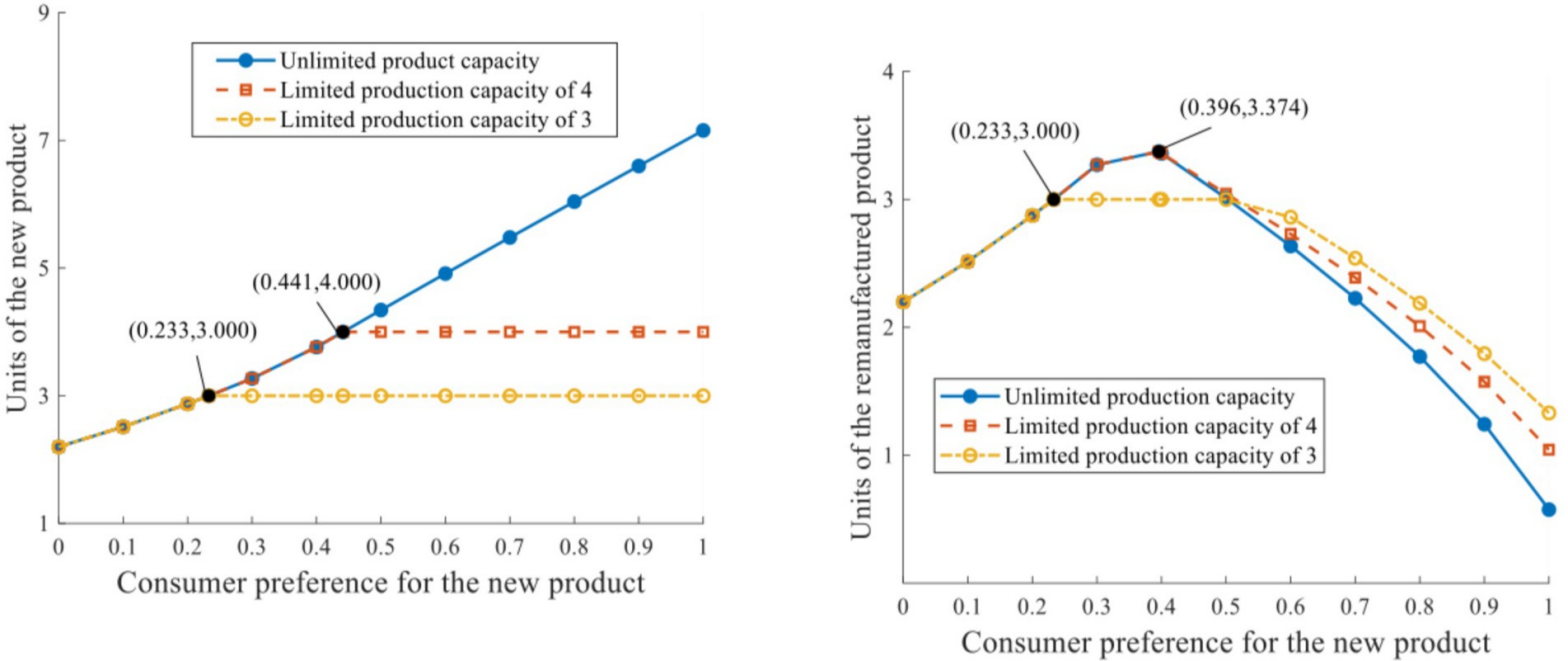
Units of products under distinct production capacities. **a.** Units of the new product. **b.** Units of the remanufactured product.

The production outputs with distinct production capacities and consumer preferences are depicted in Fig 4.

The production output of each manufacturer for the new product $q_{i}^{N*}$ increases continuously as consumer preference improves, with unlimited production capacity. If production capacity constraints exist, when $\overset{‐}{q}=3$ and *δ* = 0.233, the equilibrium production output of the new product reaches the maximum value $q_{i}^{N*}=3$. In the range of *δ*∈[0,0.233], the production output amplifies continuously as *δ* increases; while *δ* reaches 0.233 and higher, $q_{i}^{N*}$ stabilizes at 3. Similarly, when $\overset{‐}{q}=4$ and *δ* = 0.441, the equilibrium $q_{i}^{N*}$ reaches the production boundary and cannot rise any further.

The output of remanufacturer *k* for the remanufactured product is concavely related to consumer preference for the new product. The three curves in Fig 4(B) coincide when *δ*∈[0,0.233], while different tendencies occur in the range of *δ*∈(0.233,1]. The most preferable level of consumer perception for the remanufactured product is negatively related to the production capacity constraint: When $\overset{‐}{q}=4$ and *δ* = 0.396, $q_{k}^{R*}=3.374$ is the peak for remanufacturing output; when $\overset{‐}{q}=3$, the optimal $q_{k}^{R*}$ arrives at 3 when *δ* = 0.233. In addition, output of the remanufactured product is more responsive to a relaxed production capacity constraint: the curve of $\overset{‐}{q}=+\infty$, compared with those of $\overset{‐}{q}=4$ and $\overset{‐}{q}=3$, generally reduces at a faster rate when consumers are less attracted by the remanufactured product.

**Fig 5:**
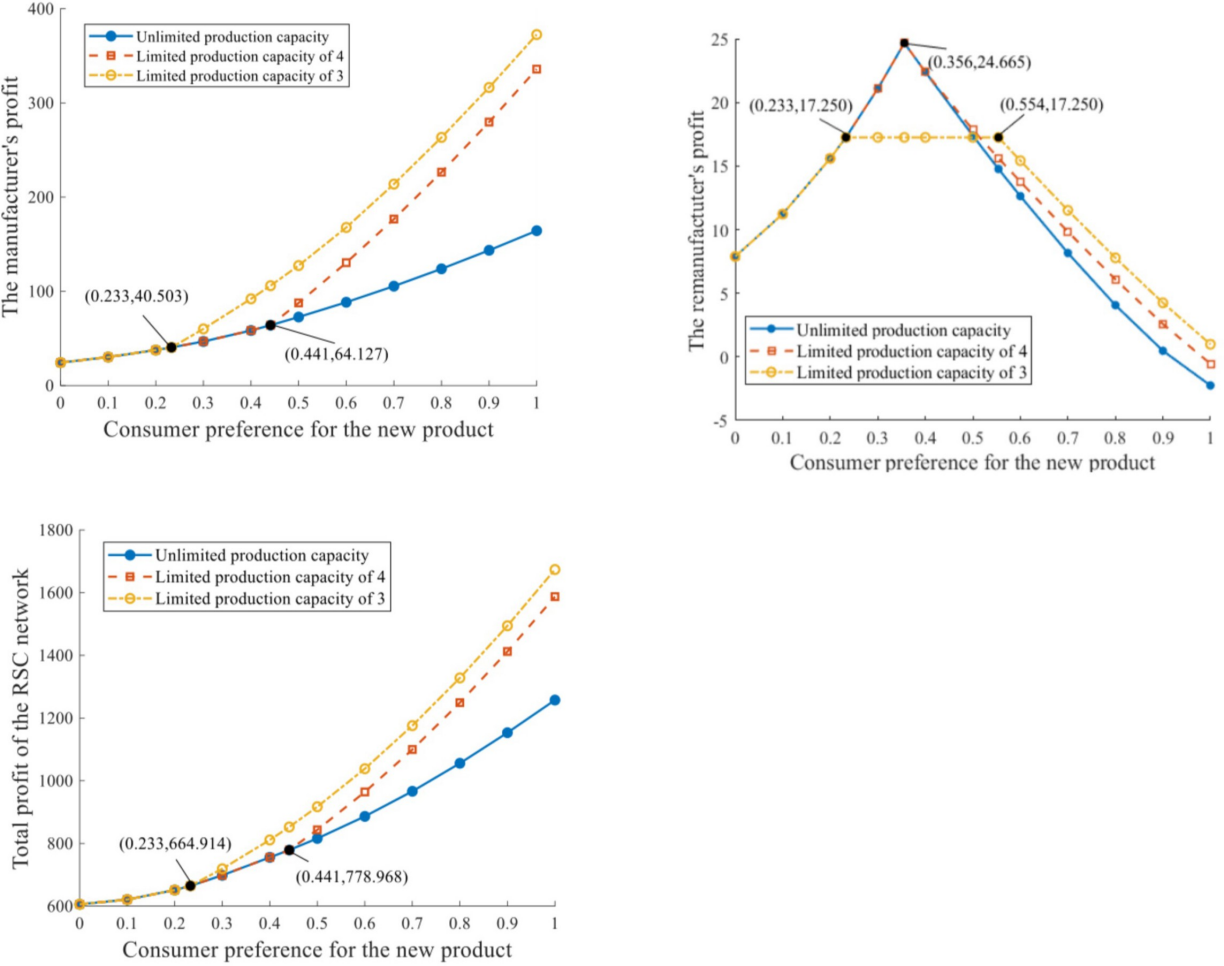
RSC network profits under distinct production capacities. **a.** Profit of the manufacturer. **b.** Profit of the remanufacturer. **c.** Profit of the RSC network.

The profits of the RSC network with distinct production capacities are depicted in Fig 5.

The manufacturer’s profit is positively related to consumer preference for the new product, and negatively impacted by the production capacity constraint. When *δ*∈ = [0,0.233], $\pi_{i}^{*}$ stays constant despite the progress of $\overset{‐}{q}$. When *δ*∈(0.233,0.441] and $\overset{‐}{q}=3$, $\pi_{i}^{*}$ increases at a faster rate while the two curves of $\overset{‐}{q}=4$ and $\overset{‐}{q}=+\infty$ still overlap. When *δ*∈(0.441,1], $\pi_{i}^{*}$ eventually reduces if given a relaxed production capacity constraint with a certain value of *δ*. The variation trend of the RSC network’s profit that responds to consumer preference is basically consistent with that of the manufacturer’s profit. In addition, the critical values of *δ* in Fig 5(C) are identical to those in Fig 5(A). Both results indicate that the manufacturer plays a pivotal role in the whole RSC network.

The remanufacturer’s profit first rises and then declines as consumer preference for the new product increases (the variation trend of remanufacturer’s profit to consumer preference under $\overset{‐}{q}=+\infty$ is similar to that under $\overset{‐}{q}=4$, and thus, we focus on only one of them). When $\overset{‐}{q}=3$ and *δ*∈[0.233,0.554], the maximum profit of the remanufacturer at equilibrium can be $\pi_{k}^{*}=17.250$. When $\overset{‐}{q}=4$, then a greater maximum value of $\pi_{k}^{*}=24.665$ can be attained. At equilibrium, the maximum attainable profit of the remanufacturer is positively related to production capacity.

**Fig 6:**
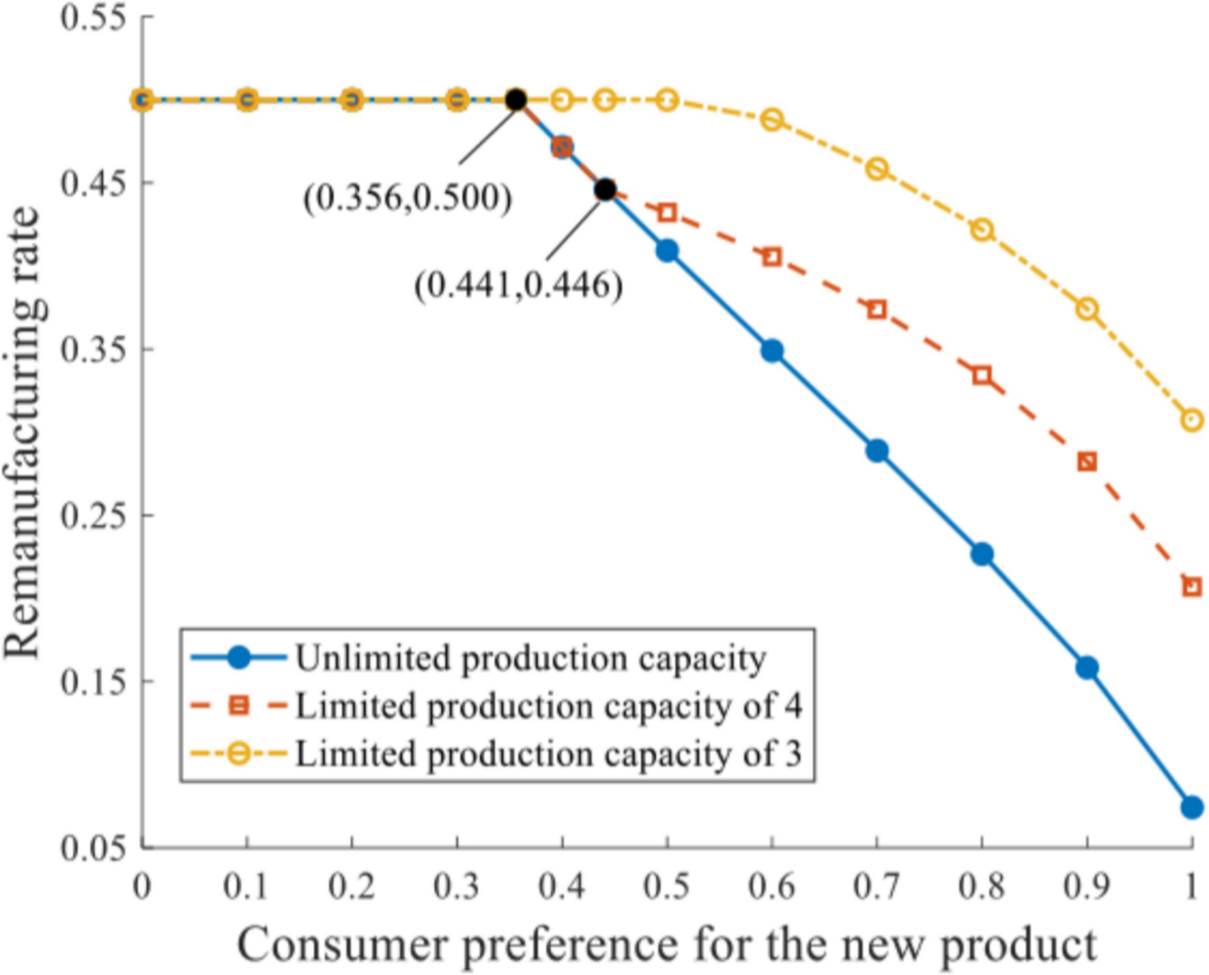
Remanufacturing rate under distinct production capacities.

The influences of production capacity constraint and consumer preference on the remanufacturing rate are depicted in Fig 6.

The remanufacturing rate at equilibrium θ^*^ is in general negatively related to *δ*. When *δ*∈[0,0.356], consumers value the remanufactured product at a relatively significant level, which invigorates the remanufacturing implementation and results in considerable remanufacturing output as well as the remanufacturing rate. θ^*^ remains at 0.5 in this range, without response to the production capacity constraints. When *δ*∈(0.356,0.441], θ^*^ reduces as *δ* increases, while its values under $\overset{‐}{q}=+\infty$ and $\overset{‐}{q}=4$ are slighter than that under $\overset{‐}{q}=3$. When *δ*∈(0.441,1], θ^*^ reduces further as the demand market values the remanufactured product even less. Given this certain range of *δ*, θ^*^ is observed to decrease at an increasing rate as production capacity magnifies.

#### Example III: Influences of price competitiveness on RSC network strategies

*Scenario III(1)*. The problem remains the same as that in Example I, except that consumer preference for the new product is set constantly at *δ* = 0.7. Moreover, the comparative price competitiveness of the new and remanufactured product are diverse, with *τ*^*N*^ = 0.3 set constantly and *τ*^*R*^ = 0.1, 0.3, 0.5 allocated diversely. Table 1 reports the new equilibrium solutions including the flows between different tiers in the RSC network, the prices charged for purchasing the products, the profits of relevant stakeholders, and the remanufacturing rate.

**Table 1:** Equilibrium solutions under distinct price competitiveness of the remanufactured product.

Entity	Variables	Price competitiveness of the remanufactured product		
		*τ*^*R*^ = 0.1	*τ*^*R*^ = 0.3	*τ*^*R*^ = 0.5
**Manufacturers**	$q_{i}^{N*}$, *i* = 1,2	5.480	5.485	5.489
	$q_{ij}^{N*}$, *i* = 1,2; *j* = 1,2	2.740	2.742	2.745
	$q_{ij}^{R*}$, *i* = 1,2; *j* = 1,2	1.114	1.307	1.485
	$p_{ij}^{N*}$, *i* = 1,2; *j* = 1,2	40.558	40.684	40.799
	$p_{ij}^{R*}$, *i* = 1,2; *j* = 1,2	22.655	25.271	27.668
	$\pi_{i}^{*}$, *i* = 1,2	105.541	106.651	107.701
**Retailers**	$p_{j}^{N*}$, *j* = 1,2	98.532	99.568	100.520
	$p_{j}^{R*}$, *j* = 1,2	62.724	68.741	74.254
	$q_{j}^{N*}$, *j* = 1,2	5.480	5.485	5.489
	$q_{j}^{R*}$, *j* = 1,2	2.227	2.615	2.970
	$\pi_{j}^{*}$, *j* = 1,2	369.542	395.742	421.938
**Remanufacturers**	$q_{k}^{R*}$, *k* = 1,2	2.227	2.615	2.970
	$q_{ki}^{R*}$, *k* = 1,2; *i* = 1,2	1.114	1.307	1.485
	$p_{ki}^{R*}$, *k* = 1,2; *i* = 1,2	17.728	20.246	22.554
	$p_{k}^{E*}$, *k* = 1,2	4.227	4.615	4.970
	$\pi_{k}^{*}$, *k* = 1,2	8.164	12.384	16.845
**Demand market**	α^*^, *k* = 1,2	4.227	4.6148	4.970
**Supply chain**	$\pi_{T}^{*}$	966.495	1029.554	1092.968
	*θ* ^*^	0.289	0.323	0.351

In general, manufacturers’ decisions, regarding production output of the new product, transaction quantities, and prices of new and remanufactured products, improve gradually, as the price competitiveness of the remanufactured product intensifies. Relevant values of remanufacturers undergo more considerable growth than those of the manufacturers. Apparently, the remanufacturers become better off as their product becomes more attractive. With the comparative increase of price competitiveness, the remanufacturing output is more significant than new product output, presenting as a more dramatic rise of $q_{k}^{R*}$ than $q_{i}^{N*}$, and therefore, the remanufacturing rate θ^*^ eventually progresses.

The transaction prices between different tiers in the RSC network produce weak and strong growth for the new and remanufactured products, respectively. Combining that production output and transaction scale of both products have also enlarged, rising prices indicate that the revenues of all stakeholders are enhanced, expressed as improvements of $\pi_{i}^{*}$, $\pi_{j}^{*}$, $\pi_{k}^{*}$, and $\pi_{T}^{*}$ at equilibrium. All of these results demonstrate that remanufacturing operation turns out to be remunerative with enhanced price competitiveness, such that the supply chain network members would be invigorated to engage in remanufacturing. Nevertheless, disutility of consumers in EOL product collection α^*^ rises as *τ*^*R*^ increases, which in some aspects, reflects consumer utility reduction due to the raised prices of both products.

**Fig 7:**
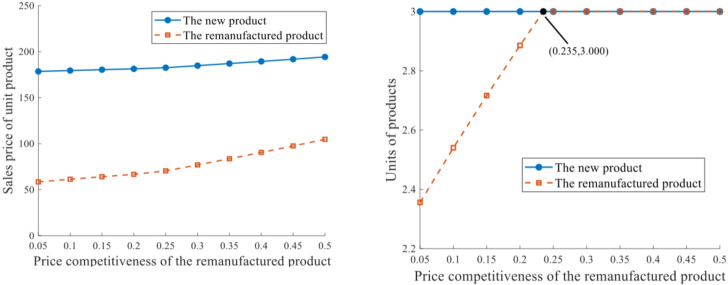
Sales price and units of products under production capacity of 3. **a.** Sales price of unit product. **b.** Units of product.

**Fig 8:**
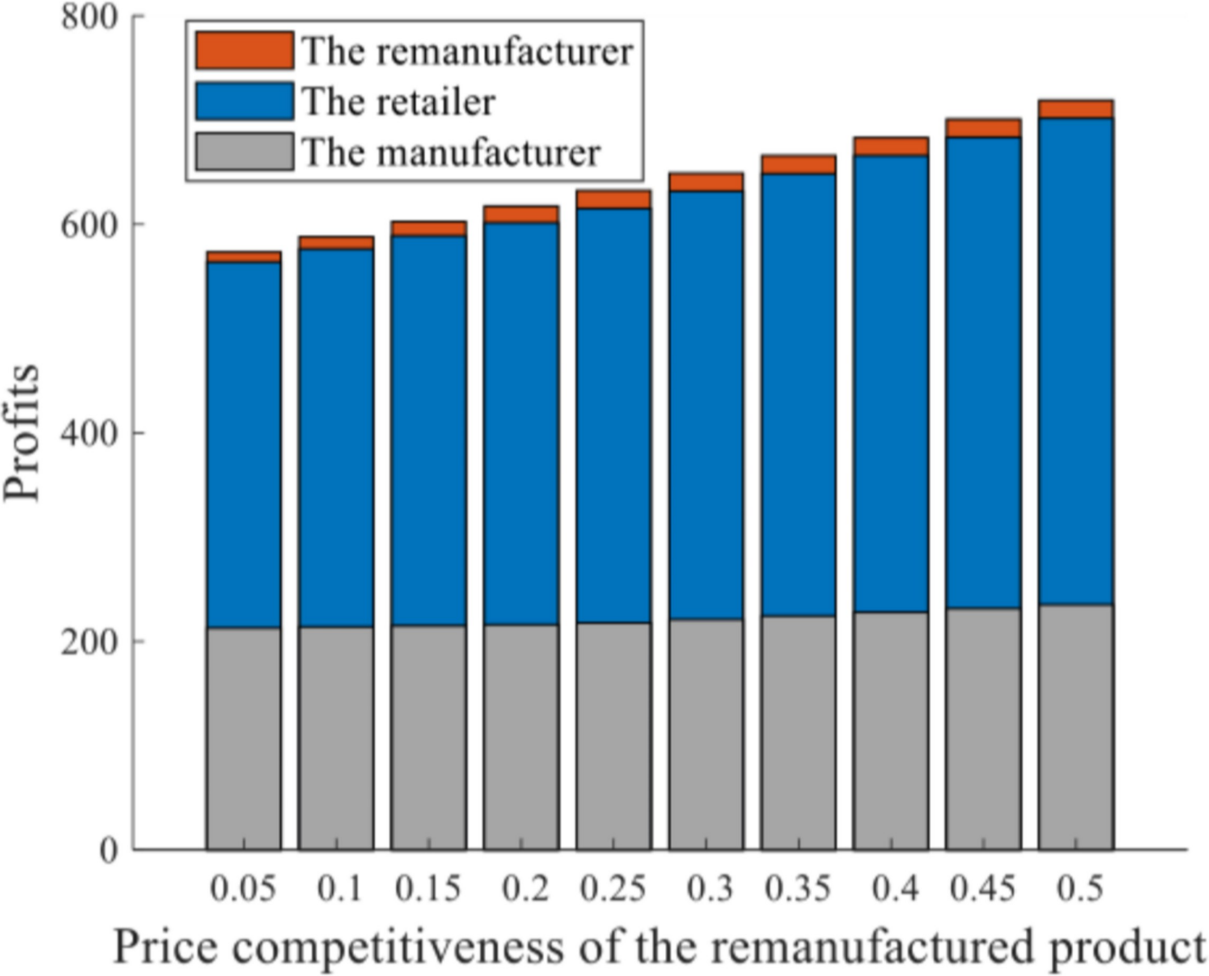
Profits of the supply chain members under production capacity of 3.

*Scenario III(2)*. This scenario generates the same situation in Scenario III(1) when comparative price competitiveness between different products varies, with a limited production capacity $\overset{‐}{q}=3$. Let *τ*^*N*^ = 0.3 be constant, and allocate various *τ*^*R*^s (τ^*R*^ ∈[0.05,0.5]). Figs 7 and 8 depict the impacts of price competitiveness on sales prices and profits of the RSC network.

The sales prices of both new and remanufactured products $p_{j}^{N*}$ and $p_{j}^{R*}$ increase with the growth of *τ*^*R*^, and the curve of $p_{j}^{R*}$ is more responsive in Fig 7(A). Units of the products, nevertheless, are greatly affected by the production capacity constraint. The equilibrium output of new product with unlimited production capacity is greater than 3 (as indicated in Table 1), however, $q_{i}^{N*}$ in Fig 7(B) remains constant at 3 despite the improvement of *τ*^*R*^, since $\overset{‐}{q}=3$ is allocated in advance. The remanufacturing output $q_{k}^{R*}$ is positively related to *τ*^*R*^ when *τ*^*R*^ ∈[0,0.235), but remains at 3 when *τ*^*R*^ ∈(0.235,1]. It can be inferred that when *τ*^*R*^ = 0.235, if all other terms are equal, there happen to be 3 units of the remanufactured product under unlimited production capacity at equilibrium. With a further increase of $\tau^{R}$, $q_{k}^{R*}$ should exceed 3, but is impeded by $\overset{‐}{q}=3$. The profits of relevant supply chain network entities as well as the whole RSC network improve with the increase of *τ*^*R*^, as Fig 8 shows. The strengthened competitiveness and acceptance of the remanufactured product ensure that remanufacturing implementation is profitable.

#### Example IV: Influences of market demand fluctuation on RSC network strategies

The functions of Example I are used in Example IV, yet consumer preference for the new product is fixed at 0.7, and demand market fluctuation is considered. Besides the upper and lower bounds of the fuzzy demand $\underset{‐}{\Delta }=\overset{‐}{\Delta }=10$, which have been adopted in Example I, additional pairs of $\underset{‐}{\Delta }=20$, $\overset{‐}{\Delta }=10$ and $\underset{‐}{\Delta }=10$, $\overset{‐}{\Delta }=20$ are allocated in this example. The new equilibrium results in a relaxed production capacity constraint (i.e. $\overset{‐}{q}=+\infty$) are reported in Table 2.

**Table 2:** Equilibrium solutions under distinct market demand fluctuations.

Entity	Variables	Variations of market demand fluctuation		
		$\underset{‐}{\Delta }=20$, $\overset{‐}{\Delta }=10$	$\underset{‐}{\Delta }=\overset{‐}{\Delta }=10$	$\underset{‐}{\Delta }=10$, $\overset{‐}{\Delta }=20$
**Manufacturers**	$q_{i}^{N*}$, *i* = 1,2	3.234	5.480	8.875
	$q_{ij}^{N*}$, *i* = 1,2; *j* = 1,2	1.612	2.740	4.434
	$q_{ij}^{R*}$, *i* = 1,2; *j* = 1,2	0.093	1.114	3.074
	$p_{ij}^{N*}$, *i* = 1,2; *j* = 1,2	26.039	40.558	62.758
	$p_{ij}^{R*}$, *i* = 1,2; *j* = 1,2	8.318	22.655	49.968
	$\pi_{i}^{*}$, *i* = 1,2	40.738	105.541	263.717
**Retailers**	$p_{j}^{N*}$, *j* = 1,2	60.930	98.532	157.560
	$p_{j}^{R*}$, *j* = 1,2	25.487	62.724	131.980
	$q_{j}^{N*}$, *j* = 1,2	3.234	5.480	8.875
	$q_{j}^{R*}$, *j* = 1,2	0.186	2.227	6.148
	$\pi_{j}^{*}$, *j* = 1,2	109.920	369.542	1217.743
**Remanufacturers**	$q_{k}^{R*}$, *k* = 1,2	0.186	2.227	6.148
	$q_{ki}^{R*}$, *k* = 1,2; *i* = 1,2	0.093	1.114	3.074
	$p_{ki}^{R*}$, *k* = 1,2; *i* = 1,2	4.462	7.723	43.212
	$p_{k}^{E*}$, *k* = 1,2	2.186	4.227	8.148
	$\pi_{k}^{*}$, *k* = 1,2	-2.922	8.164	82.045
**Demand market**	α^*^, *k* = 1,2	2.186	4.227	8.148
**Supply chain**	$\pi_{T}^{*}$	289.471	966.495	3127.010
	*θ* ^*^	0.054	0.289	0.409

Taking $\underset{‐}{\Delta }=\overset{‐}{\Delta }=10$ along with the equilibrium solutions under it as the baseline data, we note that when the lower bound of market demand grows significantly (i.e. $\underset{‐}{\Delta }=20$), the sales prices drop considerably, and profits of relevant stakeholders shrink. The product flows between different tiers in the RSC network, the remanufacturing rate, as well as consumer disutility decline simultaneously. The remanufacturer may suffer negative profit with decreased market demand as $\pi_{k}^{*}=-2.922$. On the contrary, we observe opposite results of rising prices and profits when the upper bound of market demand rises (i.e. $\overset{‐}{\Delta }=20$).

These contradictory situations bring about obvious comparisons between the optimal strategies of network entities and their utilities. Thus, it is not surprising that there is a shift in whether an RSC member should produce more or charge a higher price as market demand amplifies. In particular, the retailer that is most associated with the demand market responds actively to the market instability with dramatic fluctuations of the sales prices and profits. As the equilibrium solutions in Table 2 show, $p_{j}^{N*}$ under distinct levels of market demand varies from 60.930 to 157.560; $p_{j}^{R*}$ rises from 25.487 to 131.980; and $\pi_{j}^{*}$ grows considerably from 109.920 to 1217.743, when the upper bound of market demand rises.

#### Example V: Government regulation via fiscal instruments

In this example, we suppose consumer preference for the new product fixed at *δ* = 0.6 or *δ* = 0.7 based on the result of questionnaire survey (consumer preference for remanufactured products is 30.44% in the survey). Recall the case of Good Friend Precision Machinery, other parameters and functions of Example I are used in Example V, yet government regulation is considered. The government takes fiscal measures to achieve its balanced budget between manufacturing tax (levy imposed on a unit of new product) and remanufacturing subsidy (subsidy offered for a unit of remanufactured product). This example follows the same network structure as Example I but with varying levy functions for the manufacturers in order to focus on government budget. We vary the levy imposed on a unit of new product *f*, from 0.25 to 2.5, to deduce the corresponding sales prices and production outputs of both new and remanufactured products.

**Fig 9:**
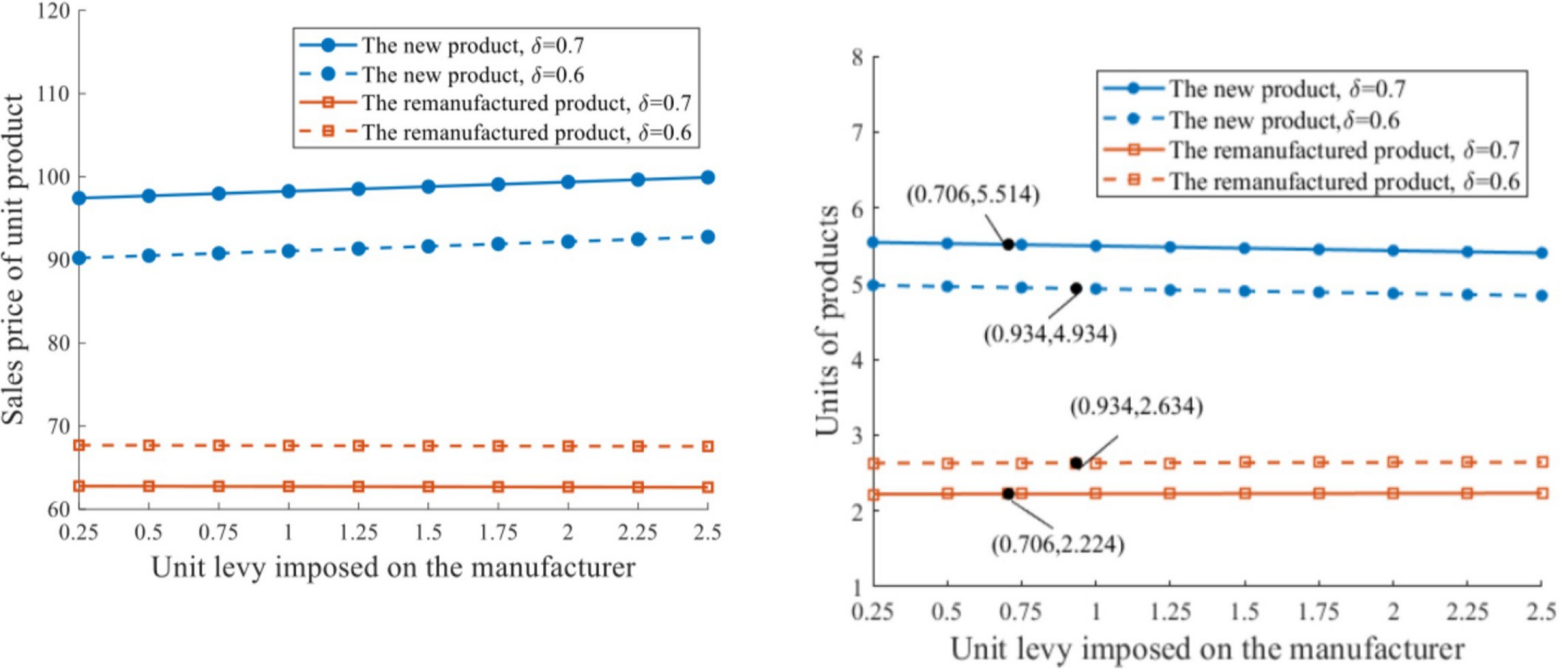
Sales price and units of products under various unit levies imposed on the manufacturer. **a.** Sales price of unit product. **b.** Units of product.

*ScenarioV(1)*. The model with unlimited production capacity $\overset{‐}{q}=+\infty$ is applied to the abovementioned new levies imposed on the new product, with the new equilibrium solutions revealed in Fig 9. The sales price of a unit of new product at equilibrium increases throughout the increase of unit levy, while that of a unit of remanufactured product decreases. Moreover, $p_{j}^{N*}$ is more responsive to the change of *f* than $p_{j}^{R*}$ is, since the levy is directly imposed on the new product.

The production outputs of the new and remanufactured products are impacted by the levy of a unit of new product negatively and positively, respectively. As for the relationship between production and consumer preference for the new product, $q_{i}^{N*}$ increases and $q_{k}^{R*}$ decreases as *δ* improves.

Given *s* = 1.75, with the increase of the levy imposed on a unit of new product, a preferable value of *f* can be determined for the government to make ends meet. (1) When *δ* = 0.7, *f* = 0.706 should be embraced for the authority to allocate, such that we obtain a pair of *f* and *s* that satisfies $fq_{i}^{N*}=sq_{k}^{R*}$. The relevant outputs of distinct products are $q_{i}^{N*}=5.514$ and $q_{k}^{R*}=2.224$. (2) When *δ* = 0.6 with improved consumer preference for the remanufactured product, a higher *f* = 0.934 needs to be charged for a unit of new product in response to the increased remanufacturing output $q_{k}^{R*}=2.634$ and decreased new production output $q_{i}^{N*}=4.934$. Equilibrium solutions of product outputs $q_{i}^{N*}$ and $q_{k}^{R*}$ under the government’s balanced budget show positive and negative relationships between *δ*, respectively. In this regard, enhanced consumer perception for the remanufactured product boost remanufacturing implementation, from the perspective of either the RSC network or government policy-making.

**Fig 10:**
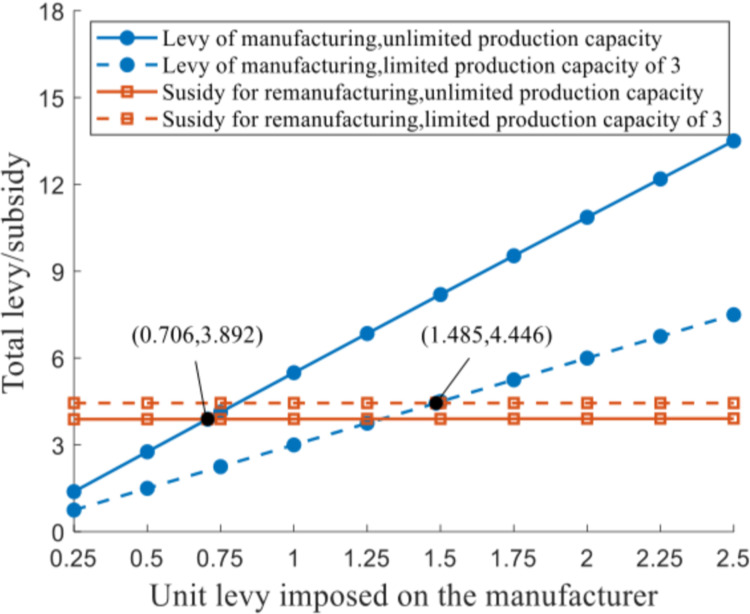
Total levy/subsidy of the product under distinct production capacities.

*Scenario V(2)*. The focus in this scenario is on government financial instruments regarding levy *f* imposed on the manufacturers for a unit of new product and subsidy *s* offered to remanufacture a unit of EOL product, in the presence of production capacity constraint $\overset{‐}{q}=3$. The variation trend of the total levy/subsidy is shown in Fig 10.

It is worth noting that the equilibrium solutions of production output of both products remain constant under $\overset{‐}{q}=3$, even though various levies are imposed on a unit of new product, presented as $q_{i}^{N*}=3$, $q_{k}^{R*}=2.540$. The total subsidy received by the remanufacturer $sq_{k}^{R*}$, in this situation, stabilizes at 4.446 when given a certain *s* = 1.75. The prerequisite for the government to achieve a balanced budget is calculated as *f* = 1.482, such that $fq_{i}^{N*}=sq_{k}^{R*}=4.446$ can be satisfied. With unlimited production capacity $\overset{‐}{q}=+\infty$, the balanced budget is attained at *f* = 0.706 and *s* = 1.75, according to which $fq_{i}^{N*}=sq_{k}^{R*}=3.892$ is fulfilled. Observation of the two pairs of *f* and *s*, as well as the balanced expenditure and revenue of the authority, indicates that capacity expansion of the manufacturer facilitates a tax cut of government to accomplish a balanced budget, thus promoting new product manufacturing and EOL product remanufacturing activities.

## Conclusions and implications

The energy crisis and environmental problems are major issues in today’s global economy, which has major challenges associated with resource conservation and environmental sustainability. Remanufacturing is infinitely superior to product reuse and recycling in realizing resource circulation, and therefore, is globally recognized by authorities and corporates. RSC network equilibrium models are productive for remanufacturing management, model formulations and solutions have been active areas of environmental supply chain research owing to the applicability of remanufacturing in practice. We conclude the results and managerial implications of significant references for enhancing RSC network benefits as well as improving government financial regulations.

### Concluding remarks

This paper captures product heterogeneity and variance of consumer preference toward differentiated products in an RSC network. Considering that the government has a direct stake in curbing negative externalities generated by new product production and consumption, and has the power to take corrective measures, we complement a government’s attempt to achieve a balanced budget between a levy imposed on manufacturing and a subsidy offered for remanufacturing. Production capacity constraint and fuzzy demand are considered to advance the modelling analysis and pragmatic application of the RSC network model. Based on variational inequality theory, we obtain and analyse the optimal behaviour of RSC members as well as the government by LQP-PC algorithm. The numerical examples based on a real case and questionnaire survey further apply the RSC network model with consumer preference, production capacity constraint and fiscal regulation. We conclude the main results as follows.

- For the influences of consumer preference on RSC network decisions, if the remanufactured product has not gained much recognition and consumers value the new product highly, remanufacturing is unprofitable. With the enhancement of consumer preference for the remanufactured product, pricing for it would be equivalent to or even exceed that of the new product. However, if consumers have an excessive penchant for the remanufactured product, output and sales volume of the new product reduce, which would eventually curb remanufacturing since new product is the origin of EOL product for remanufacturing. In general, an upper-middle level of consumer perception and acceptance for remanufacturing is the most favourable scenario for remanufacturing.
- For the influence of price competitiveness of the remanufactured product, the enhancement of price competitiveness basically improves the benefits of the RSC network members, but harms consumer welfare. Since production output, transaction quantities, and prices of new and remanufactured products improve as price competitiveness of the remanufactured product intensifies, remanufacturing turns out to be remunerative, such that the RSC network members would be invigorated to engage in remanufacturing. Yet, consumer welfare reduces and disutility rises due to improved prices and transaction quantities of both products.
- For the influence of market demand fluctuation, the flows of products between different tiers, the prices for purchasing the products, the profits of the RSC network, and the remanufacturing rate basically improve as the upper bound of fuzzy demand expands. In particular, the retailer responds more actively than the manufacturer and remanufacturer, to market demand fluctuation, since it is most associated with the market, with dramatic variances of the sales prices and profits.
- RSC members have to follow the fiscal policy for remanufacturing to maintain well-run operations. Since the manufacturing tax is directly imposed on the new product, increased levy imposed on a unit of new product would decrease output of this kind of product. Thus, given the subsidy offered for a unit of remanufactured product, outputs of the new and remanufactured products are negatively and positively impacted by unit levy. When remanufacturing industry is just emerging, a high tax will be imposed on the manufacturer to accumulate remanufacturing subsidy, from the perspective of government’s attempt to make ends meet and promote remanufacturing industry in the short term. As the remanufacturing industry develops and consumer environmental awareness enhances, a relatively lower tax would facilitate to expand output of both products, thus progressing remanufacturing industry in the long run.
- For the influences of production capacity constraint on RSC network decisions, output of the remanufactured product increases as production capacity expands, which is a logic consequence of increased output of new product that is the origin of EOL product for remanufacturing. Meanwhile, output of the remanufactured product is less responsive to consumer preference under stricter production capacity constraint, which is in line with the case of Good Friend Precision Machinery. Output of its remanufactured machine tools is constant despite demand fluctuation and consumer preference variance due to capacity and technology constraint.

### Managerial implications

Combining the concluding remarks with the background of remanufacturing industry, we provide managerial implications from three perspectives of enterprises’ remanufacturing operation, government financial regulation, and consumer awareness enhancement.

#### (1) Enterprises’ remanufacturing operations

The remanufacturer undertakes remanufacturing activities when receiving orders from its client manufacturers, and then deliver the remanufactured product to manufacturers. Thus, the case in this paper is a typical example of outsourced remanufacturing mode which is preferred by many OEMs in the US and Europe. In practice, remanufacturing industry in China is still at an initial stage, where manufacturers with advanced technology and capability of remanufacturing normally remanufacture EOL products by themselves. To be specific, firms such as Sany Construction Machinery Remanufacturing Cooperation (SCMRC), Weichai Remanufacturing Group Ltd. (WRG), and Jinan Fuqiang Power Ltd., which are selected by the Chinese government as leading remanufacturing pilot enterprises, have been engaged in self-remanufacturing with a good reputation [55]. Most 3PRs like Good Friend Precision Machinery, by contrast, are small-scale firms with limited production capacity, capital and technology to fulfil remanufacturing orders. Besides, output of its remanufactured machine tools is practically constant despite demand fluctuation and consumer preference variance due to limited production capacity and technology. The results hold in Example II that output of the remanufactured product is less responsive to consumer preference under stricter production capacity constraint.

Outsourced remanufacturing boosts long-term development of remanufacturing, and accelerates the process of integrity and maturity of the industrial chain. In this point, manufacturers are suggested to entrust the leading remanufacturing pilot firms to remanufacture EOL products regarding used automobile, machine tool and construction machinery.

#### (2) Government financial regulation

In the case of Good Friend Precision Machinery, unit subsidy of 1750 CNY (~$271) is offered according to actual situation. We scale it down in proportion and use *s* = 1.75 in the proposed model. When remanufacturing industry is still at the initial stage, consumers value the new product at a high level as *δ* = 0.7, and the remanufacturer faces a production capacity constraint $\overset{‐}{q}=3$. The tax imposed on new product manufacturing can be calculated as *f* = 1.482 to obtain a balanced budget. Then given the government financial measures, equilibrium strategies of sales prices of new and remanufactured products are: $p_{i}^{N}=179.53$,$p_{k}^{R}=61.38$. We note that the tax imposed on the manufacturer *f* is relatively high, and sales price of the new product is much higher than that of the remanufactured product.

As the remanufacturing industry expands continuously and enterprises enhance production capacity as well as remanufacturing related technologies, $\overset{‐}{q}=+\infty$ in the case is considered for the government to adjust financial measures. By this time, we have the equilibrium solutions as: *f* = 0.706, $p_{i}^{N}=97.93$, $p_{k}^{R}=62.76$. Sales price of the remanufactured product is 64.09% of that of the new product, which turns out to be reasonable and practical.

In the proposed model and numerical examples, we suppose the government regulates remanufacturing operations via tax and subsidy measures. In practice, the Chinese government intervenes remanufacturing implementations via subsidy measures alone, while tax policy that imposed on the manufacturer to collect remanufacturing funds is not executed. Considering that Chinese remanufacturing industry is just emerging, we regard such a subsidy measure as government support for remanufacturing enterprises, so it is applicable in the short term to expand remanufacturing output and invigorate remanufacturing activities. In the long term, however, the government should carry out rational measures to alleviate financial pressure. In addition to providing subsidy for remanufactured product remanufacturing, levy should also be imposed on new product manufacturing to collect fund for recycling and remanufacturing. A combined tax and subsidy mechanism would be helpful to achieve a balanced budget.

#### (3) Consumer preference

Recall the case of Good Friend Precision Machinery, the price of remanufactured product would exceed that of the new product when consumer preference is more than 60.4%, and the most preferable level of consumer preference for the remanufacturer to achieve maximum profit is 64.6% (as is shown in Fig 2). Nevertheless, questionnaire survey on consumer recognition of the remanufactured product shows that consumers are less attracted by the remanufactured product than the new one (consumer preference for the remanufactured product is 30.44%). A lack of consumer preference for the remanufactured product also exacerbates demand uncertainty, which might count against profiting and progressing of remanufacturing industry.

Besides, when consumers value the remanufactured product at a low level, price competitiveness of the new product would be higher than that of the remanufactured one (as is reflected as *τ*^*N*^ = 0.3 and *τ*^*R*^ = 0.1 in the examples), which indicates that even if both products share the same price, consumers will still purchase the new product. As a result, the remanufactured product will be underprized, which discourages enterprises to collect and remanufacture EOL product since they are facing the risk of losing profits from remanufacturing. The remanufacturing rate eventually turns out to be insignificant as Fig 6 shows.

Currently, Chinese remanufacturing is still at an initial stage where public recognition of remanufactured products is relatively insufficient, making it arduous for enterprises to make profits from remanufacturing. In this aspect, the government and enterprises should react immediately to enhance publicity and popularization of remanufacturing related information to consumers. Laws and regulations enacted by the government are supposed to be disseminated effectively to enhance consumer awareness of remanufacturing operations. Internet, television and other mass media are useful for firms to introduce their remanufactured products with environmentally friendly attributes to the public.

With the enhancement of consumer environmental awareness, acceptance and preference for the remanufactured product would be improved. Increasing sales price of the remanufactured product at this moment will boost remanufacturer’s profit, thus invigorating remanufacturing implementations. Excessive preference for the remanufactured product, however, goes against sustainable promotion of remanufacturing industry in the long run. Since the new and remanufactured products are distinct but substitutable, exorbitant preference for the remanufactured product indicates deficient preference for the new product which is the origin of EOL products for remanufacturing. Thus, reduced output and sales volume eventually curbed remanufacturing implementation along with output of remanufactured product. Generally, an upper-middle level of consumer preference for the remanufactured product, 64.6% in the specific case, is the most favourable scenario for conducting remanufacturing activities, especially from the perspective of the remanufacturer.

There is still room for future research. First, we limit the model to the static case, that is, product life cycle is absent in the model, while multi-period and adaptive scenarios deserve attention in the future. Second, we assume that consumer preference for the remanufactured product varies, which is only a preliminary step in understanding what happens in the real world. Empirical studies are needed to understand consumer perceptions of specific remanufactured products.

## Supporting information

S1 Appendix Notations. (PDF)

S2 Appendix Expected profit of the retailer and its qualitative property. (PDF)

S3 Appendix Proof of Theorem 1. (PDF)

S4 Appendix Endogenous variables. (PDF)

S5 Appendix Qualitative studies. (PDF)

S6 Appendix Questionnaire survey and field research. (PDF)

S7 Appendix Algorithm. (PDF)
