# Supplementary material for: A remanufacturing supply chain network with differentiated new and remanufactured products considering consumer preference, production capacity constraint and government regulation
Source: PLoS One. 2023 Aug 10;18(8):e0289349. doi: 10.1371/journal.pone.0289349 (PMC10414650; doi:10.1371/journal.pone.0289349)
Supplement: S1 Appendix — (PDF) [file pone.0289349.s001.pdf]

S1 Appendix. Notations.

The network consists of *m* manufacturers, each one denoted by *i*; $i=1,\cdots,m$, *n* retailers, with a typical one denoted by *j*; $j=1,\cdots,n$, *o* remanufactures, each one defined by *k*; $k=1,\cdots,o$, and one typical demand market. *N*, *R*, *E* indicate the new product, remanufactured product and EOL-product, respectively. Relevant variables and parameters are listed as follows.

Decision variables:

q_i^N : Production output of new product processed and manufactured by manufacturer *i*. Group these variables into an *m*-dimensional column vector Q_m^N.

q_k^R : Production quantity of remanufactured product processed and remanufactured by remanufacturer *k*. Group these variables into an *o*-dimensional column vector Q_o^R.

q_{ij}^N : Transaction quantity of new product between manufacturer *i* and retailer *j*. Group these variables into an *mn*-dimensional column vector Q_{mn}^N.

q_{ij}^R : Transaction quantity of remanufactured product between manufacturer *i* and retailer *j*. Group these variables into an *mn*-dimensional column vector Q_{mn}^R.

q_j^N : Order volume of new product of retailer *j*, $q_j^N=\sum_{i=1}^{m} q_{ij}^N$. Group these variables into an *n*-dimensional column vector Q_n^N.

q_j^R : Order volume of remanufactured product of retailer *j*, $q_j^R=\sum_{i=1}^{m} q_{ij}^R$. Group these variables into an *n*-dimensional column vector Q_n^R.

q_{ki}^R : Transaction quantity of remanufactured product between remanufacturer *k* and manufacturer *i*. Group these variables into an *om*-dimensional column vector Q_{om}^R.

q_k^E : Transaction quantity of EOL-product between the demand market and remanufacturer *k*. Group these variables into an *o*-dimensional column vector Q_o^E.

p_j^N : Sale price of unit new product by retailer *j*. Group these variables into an *n*-dimensional column vector P_n^N.

p_j^R : Sale price of unit remanufactured product by retailer *j*. Group these variables into an *n*-dimensional column vector P_n^R.

Endogenous variables:

p_{ij}^N : Transition price of unit new product between manufacturer *i* and retailer *j.*

p_{ij}^R : Transition price of unit remanufactured product between manufacturer *i* and retailer *j.*

p_{ki}^R : Transition price of unit remanufactured product between remanufacturer *k* and manufacturer *i.*

p_k^E : Recycling price of unit EOL-product between remanufacturer *j* and the demand market.

Other parameters:

$\overline{q}$: Production capacity of new product processed and manufactured by manufacturer *i.*

τ^N : Price competitive intensity of new product to remanufactured product.

τ^R : Price competitive intensity of remanufactured product to new product.

δ : Consumer preference for the new product, and $(1-\delta)$ stands for consumer preference for the remanufactured product.

$c_i^N\left(Q_m^N\right)$: Manufacturing cost of new product by manufacturer *i.*

$c_k^R\left(Q_o^R\right)$: Remanufacturing cost of remanufactured product by remanufacturer *i.*

$w_j\left(Q_n^N, Q_n^R\right)$: Sales and transaction costs of new and remanufactured product by retailer *j.*

$w_{ij}\left(q_{ij}^N, q_{ij}^R\right)$: Transaction cost between manufacturers and retailers, which is assumed by manufacturers.

$w_k\left(q_k^E\right)$: Transaction cost between the demand market and remanufacturers, which is

assumed by remanufacturers.

$w_{ki}\left(q_{ki}^{R}\right)$: Transaction cost between remanufacturers and manufacturers, which is assumed by remanufacturers.

$d_{j}^{N}=d_{j}^{N}\left(p_{j}^{N},p_{j}^{R},\tau^{N},\delta\right)$: Fuzzy demand of the market to the new product of retailers.

$d_{j}^{R}=d_{j}^{R}\left(p_{j}^{N},p_{j}^{R},\tau^{R},\delta\right)$: Fuzzy demand of the market to the remanufactured product of retailers.

$\alpha_{k}^{E}\left(Q_{o}^{E}\right)$: Disutility of consumers in EOL-product collection.

$f_{i}^{N}\left(q_{i}^{N}\right)$: Levy imposed on unit new product of manufacturer *i* by government for remanufacturing, where *f* denotes the unit levy.

$s_{k}^{R}\left(q_{k}^{R}\right)$: Subsidy provided for unit remanufactured product of remanufacturer *k* by government, where *s* denotes the unit subsidy.

M: Market demand space of both new and remanufactured products.

$\overline{\Delta}$: Upper limit of market demand fluctuation.

$\underline{\Delta}$: Inferior limit of market demand fluctuation.
