# Supplementary material for: A remanufacturing supply chain network with differentiated new and remanufactured products considering consumer preference, production capacity constraint and government regulation
Source: PLoS One. 2023 Aug 10;18(8):e0289349. doi: 10.1371/journal.pone.0289349 (PMC10414650; doi:10.1371/journal.pone.0289349)
Supplement: S2 Appendix — (PDF) [file pone.0289349.s002.pdf]

S2 Appendix. Expected profit of the retailer and its qualitative property.

According to (6), we have the expected profit of the retailer j expressed as

$$E\left[\pi_j\right] = p_j^N \cdot E\left[\min\left(d_j^N, v_j^N\right)\right] + p_j^R \cdot E\left[\min\left(d_j^R, v_j^R\right)\right] - \sum_{i=1}^{m}\left[q_{ij}^N p_{ij}^N + q_{ij}^R p_{ij}^R\right] - w_j\left(q_j^N, q_j^R\right)$$

(S2.1)

where $E\left[\min\left(d_j^N, q_j^N\right)\right] = q_j^N - \int_{\underline{d}_j^N}^{q_j^N}\left(q_j^N - x\right) d\sigma_j^N(x)$ and $E\left[\min\left(d_j^R, q_j^R\right)\right] = q_j^R - \int_{\underline{d}_j^R}^{q_j^R}\left(q_j^R - x\right) d\sigma_j^R(x)$.

The proof of retailer's expected profit is completed.

From (9), we obtain the first-order and second derivatives of $E\left[\pi_j\right]$ to the transaction quantity of new product q_{ij}^o as

$$\frac{\partial E\left[\pi_j\right]}{\partial q_{ij}^N} = p_j^N - p_j^N \sigma_j^N - p_{ij}^N - \frac{\partial w_j}{\partial q_{ij}^N} \qquad \text{(S2.2)}$$

$$\frac{\partial E^2\left[\pi_j\right]}{\partial\left(q_{ij}^N\right)^2} = -\frac{\partial^2 w_j}{\partial\left(q_{ij}^N\right)^2} \qquad \text{(S2.3)}$$

All variables are nonnegatively defined and we assume the transaction cost is convex and continuously differentiable. Hence, $E\left[\pi_j\right]/\partial q_{ij}^N < 0$ and $E^2\left[\pi_j\right]/\partial\left(q_{ij}^N\right)^2 < 0$ can be achieved. We conclude that $E\left[\pi_j\right]$ is the concave function of $\left\{q_{1j}^o, \cdots q_{ij}^o, \cdots q_{mj}^o\right\}$. The same result holds for the transaction quantity of the remanufactured product, that is, $E\left[\pi_j\right]$ is the concave function of $\left\{q_{1j}^r, \cdots q_{ij}^r, \cdots q_{mj}^r\right\}$.
