# Supplementary material for: A remanufacturing supply chain network with differentiated new and remanufactured products considering consumer preference, production capacity constraint and government regulation
Source: PLoS One. 2023 Aug 10;18(8):e0289349. doi: 10.1371/journal.pone.0289349 (PMC10414650; doi:10.1371/journal.pone.0289349)
Supplement: S3 Appendix — (PDF) [file pone.0289349.s003.pdf]

S3 Appendix. Proof of Theorem 1.

For easy reference in subsequent sections, variational inequality problem (20) can be rewritten in a standard variational inequality formgiven by: determine $\left(Q_m^{N*},Q_o^{R*},Q_{mn}^{N*},Q_{mn}^{R*},Q_{om}^{R*},Q_o^{E*},Q_n^{N*},Q_n^{R*},P_n^{N*},P_n^{R*},\ \lambda^*,\gamma^*,\mu^*,\eta^*,\varepsilon^*,\beta^*,\rho^*,\xi^*\right)\in K$, satisfying

$$\left\langle F\left(X^*\right),X-X^*\right\rangle\geq 0,\quad \forall X\in K \tag{S3.1}$$

where $X\equiv\left(Q_m^{N*},Q_o^{R*},Q_{mn}^{N*},Q_{mn}^{R*},Q_{om}^{R*},Q_o^{E*},Q_n^{N*},Q_n^{R*},P_n^{N*},P_n^{R*},\lambda^*,\gamma^*,\mu^*,\eta^*,\varepsilon^*,\beta^*,\rho^*,\xi^*\right)$ and $F=\left\{\left(F_i^1,F_k^2,F_{ij}^3,F_{ij}^4,F_{ki}^5,F_k^6,F_j^7,F_j^8,F_j^9,F_j^{10},F_i^{11},F_i^{12},F_i^{13},F_j^{14},F_j^{15},F_k^{16},F_k^{17},F^{18}\right)\right\}_{i=1,\cdots,m;\,j=1,\cdots n;k=1,\cdots,o}$

, and the terms of F correspond to the terms preceding the multiplication signs in inequality (20). K is the feasible set,

$K=\left\{\left(Q_m^{N*},Q_o^{R*},Q_{mn}^{N*},Q_{mn}^{R*},Q_{om}^{R*},Q_o^{E*},Q_n^{N*},Q_n^{R*},P_n^{N*},P_n^{R*},\lambda^*,\gamma^*,\mu^*,\eta^*,\varepsilon^*,\beta^*,\rho^*,\xi^*\right)\right|$

$q_i^N\geq 0,q_k^R\geq 0,q_{ij}^N\geq 0,q_{ij}^R\geq 0,q_{ki}^R\geq 0,q_k^E\geq 0,q_j^N\geq 0,q_j^R\geq 0,p_j^N\geq 0,p_j^R\geq 0,\lambda_i\geq 0,\gamma_i\geq 0,\mu_i\geq 0,\eta_j\geq 0,$

$\varepsilon_j\geq 0,\beta_k\geq 0,\rho_k\geq 0,\xi\geq 0\left|\forall i,j,k\right\}$ must be closed and convex.

With some algebraic manipulation and adding (5), (10), (14) and (19), it follows that the result is inequality (20). If we add $\left(-p_{ij}^{N*}+p_{ij}^{N*}\right)$ to the third set of brackets and $\left(-p_k^{E*}+p_k^{E*}\right)$ to the sixth set of brackets to the variational inequality (20), inequality (20) will not change since the value of these additions is simply zero. However, the variational inequality formulation (25) with additional terms can be rewritten as the summation of variational inequalities (5), (10), (14) and (19). The proof is completed.
