# Supplementary material for: A remanufacturing supply chain network with differentiated new and remanufactured products considering consumer preference, production capacity constraint and government regulation
Source: PLoS One. 2023 Aug 10;18(8):e0289349. doi: 10.1371/journal.pone.0289349 (PMC10414650; doi:10.1371/journal.pone.0289349)
Supplement: S4 Appendix — (PDF) [file pone.0289349.s004.pdf]

S4 Appendix. Endogenous variables.

Note that the variables in the model, which can be determined from the solution of either variational inequality (20) or (S3.1) are: the equilibrium new product output given by Q_m^N, the equilibrium remanufactured products output denoted by Q_o^R, the equilibrium new and remanufactured products transaction quantities from the manufactures to the retailers expressed by Q_{mn}^N and Q_{mn}^R, the equilibrium remanufactured product transaction quantities from the third-party remanufacturer to the manufacturer denoted by Q_{om}^R, the equilibrium EOL-collection amount from consumers to the remanufactures given by Q_o^E, the equilibrium order volumes of new and remanufactured products expressed by Q_n^N and Q_n^R, as well as the equilibrium demand prices P_n^N and P_n^R. Now we can retrieve the equilibrium prices p_{ij}^N and p_{ij}^R that manufacturers charge retailers for new and remanufactured products respectively, p_{ki}^R associated with the price that remanufacturers charge manufacturers for the remanufactured product and p_k^E associated with the EOL-collection price between remanufacturers and consumers.

After solving the variational inequality (20), we can find the optimal values of the Lagrange multipliers: γ_i^*, μ_i^*, η_j^* and ε_j^*. Then from (5) the transaction prices between manufacturers and retailers can be recovered for any *i* and *j* such that if $q_{ij}^{N*} > 0$ and $q_{ij}^{R*} > 0$, we set

$$p_{ij}^{N*} = \frac{\partial w_{ij}^*}{\partial q_{ij}^N} + \gamma_i^* \tag{S4.1}$$

$$p_{ij}^{R*} = \frac{\partial w_{ij}^*}{\partial q_{ij}^R} + \mu_i^* \tag{S4.2}$$

or, equivalently to (cf. (10))

$$p_{ij}^{N*} = -\frac{\partial w_j^*}{\partial q_{ij}^N} + \eta_j^* \tag{S4.3}$$

$$p_{ij}^{R*} = -\frac{\partial w_j^*}{\partial q_{ij}^R} + \varepsilon_j^* \tag{S4.4}$$

Also, from (14) it follows that if $q_{ki}^{R*} > 0$, then

$$p_{ki}^{R*} = \frac{\partial w_{ki}^*}{\partial q_{ki}^R} + \beta_k^* \tag{S4.5}$$

From (19), if the optimal quantity of EOL-collection $q_k^{E*} > 0$, then we can set the buy-back price

$$p_k^{E*} = \alpha_k^E \left(Q_o^{E*}\right) + \xi^* \tag{S4.6}$$
