# Supplementary material for: A remanufacturing supply chain network with differentiated new and remanufactured products considering consumer preference, production capacity constraint and government regulation
Source: PLoS One. 2023 Aug 10;18(8):e0289349. doi: 10.1371/journal.pone.0289349 (PMC10414650; doi:10.1371/journal.pone.0289349)
Supplement: S5 Appendix — (PDF) [file pone.0289349.s005.pdf]

S5 Appendix. Qualitative studies.

Here, we provide some qualitative properties of the solutions to variational inequality (20), in particular, the existence results.

The feasible set underlying the variational inequality problem (20) is not compact. Therefore, we cannot derive existence of a solution simply from the assumption of continuity of the functions. However, by imposing a rather weak condition, we can guarantee the existence of a solution pattern. Let

$$\begin{aligned} K_b = \Big\{ & \left(Q_m^N, Q_o^R, Q_{mn}^N, Q_{mn}^R, Q_{om}^R, Q_o^E, Q_n^N, Q_n^R, P_n^N, P_n^R, \lambda, \gamma, \mu, \eta, \varepsilon, \beta, \rho, \xi\right) \Big| 0 \le Q_m^N \le b_1, \\ & 0 \le Q_o^R \le b_2, 0 \le Q_{mn}^N \le b_3, 0 \le Q_{mn}^R \le b_4, 0 \le Q_{om}^R \le b_5, 0 \le Q_o^E \le b_6, 0 \le Q_n^N \le b_7, \\ & 0 \le Q_n^R \le b_8, 0 \le P_n^N \le b_9, 0 \le P_n^R \le b_{10}, 0 \le \lambda \le b_{11}, 0 \le \gamma \le b_{12}, 0 \le \mu \le b_{13}, \\ & b_{14} \le \eta \le b_{15}, b_{16} \le \varepsilon \le b_{17}, 0 \le \beta \le b_{18}, 0 \le \rho \le b_{19}, 0 \le \xi \le b_{20} \Big\} \end{aligned} \quad \text{(S5.1)}$$

where $b = (b_1, \ldots b_{20}) \ge 0$. These conditions ensure that K_b is a bounded and closed convex subset of $R^{A_1 + A_2 + A_3 + A_4}$ where $A_1 = 4m + 2mn + om$, $A_2 = 2mn + 4n$, $A_3 = 2n + o + 1$, and $A_4 = om + 4o$. Hence, the following variational inequality admits at least one solution $X^b \in K_b$ since K_b is compact and F is continuous.

$$\left\langle F\left(X^b\right), X - X^b \right\rangle \ge 0, \quad X^b \in K_b \quad \text{(S5.2)}$$

Following Theorem 1.5 in Nagurney [1], Lemma 1 can hold as:

Lemma 1. Variational inequality (S1.1) admits a solution if and only if there exists a $b > 0$ such that variational inequality (S5.2) admits a solution in K_b with

$$\begin{aligned} & Q_m^N \le b_1, Q_o^R \le b_2, Q_{mn}^N \le b_3, Q_{mn}^R \le b_4, Q_{om}^R \le b_5, Q_o^E \le b_6, Q_n^N \le b_7, Q_n^R \le b_8, P_n^N \le b_9, P_n^R \le b_{10}, \\ & \lambda \le b_{11}, \gamma \le b_{12}, \mu \le b_{13}, b_{14} \le \eta \le b_{15}, b_{16} \le \varepsilon \le b_{17}, \beta \le b_{18}, \rho \le b_{19}, \xi \le b_{20} \end{aligned} \quad \text{(S5.3)}$$

Under the conditions in Theorem 2 given below, it is possible to construct a b set large enough so that the restricted variational inequality (S5.2) will meet the boundedness condition (S5.3)

and, hence, the existence of a solution to the original variational inequality (20) is guaranteed under **Lemma 1**.

Theorem 2. Existence

Suppose that there exist positive constants *S*, *T*, *W*, such that:

$$\frac{\partial c_i^N}{\partial q_i^N}+\frac{\partial f_i^N}{\partial q_i^N}\geq W\,,\quad \forall Q_m^N \text{ with } q_i^N\geq S\,,\quad \forall i \tag{S5.4}$$

$$\frac{\partial c_k^R}{\partial q_k^R}-\frac{\partial s_k^R}{\partial q_k^R}\geq W\,,\quad \forall Q_o^R \text{ with } q_k^R\geq S\,,\quad \forall k \tag{S5.5}$$

$$\frac{\partial w_{ij}}{\partial q_{ij}^N}+\frac{\partial w_j}{\partial q_{ij}^N}\geq W\,,\quad \forall Q_{mn}^N \text{ with } q_{ij}^N\geq S\,,\quad \forall i,j \tag{S5.6}$$

$$\frac{\partial w_{ij}}{\partial q_{ij}^R}+\frac{\partial w_j}{\partial q_{ij}^R}\geq W\,,\quad \forall Q_{mn}^R \text{ with } q_{ij}^R\geq S\,,\quad \forall i,j \tag{S5.7}$$

$$\frac{\partial w_{ki}}{\partial q_{ki}^R}\geq W\,,\quad \forall Q_{om}^R \text{ with } q_{ki}^R\geq S\,,\quad \forall k,i \tag{S5.8}$$

$$\alpha_k^E\left(Q_o^E\right)+\frac{\partial w_k}{\partial q_k^E}\geq W\,,\quad \forall Q_o^E \text{ with } q_k^E\geq S\,,\quad \forall k,i \tag{S5.9}$$

$$E\left[d_j^N\right]\leq S\,,\quad \forall P_n^N \text{ with } p_j^N>T\,,\quad \forall j \tag{S5.10}$$

$$E\left[d_j^R\right]\leq S\,,\quad \forall P_n^R \text{ with } p_j^R>T\,,\quad \forall j \tag{S5.11}$$

Then variational inequality (20) as well as (S2.1) admit at least one solution.

Proof. Follows from **Lemma 1**. See also the proof of existence in Nagurney [1] and Nagurney et al. [2].

References

[1] Nagurney A. Network economics: a variational inequality approach. 2nd and revised ed. Boston, MA: Kluwer Academic Publishers; 1999.

[2] Nagurney A, Yu M, Floden J. Fashion supply chain network competition with ecolabeling. In Choi, T. & Cheng, T. (Eds.) Sustainable fashion supply chain management. In Springer series in supply chain management: 1 (pp. 61-84). Springer; 2015.
