# Supplementary material for: A remanufacturing supply chain network with differentiated new and remanufactured products considering consumer preference, production capacity constraint and government regulation
Source: PLoS One. 2023 Aug 10;18(8):e0289349. doi: 10.1371/journal.pone.0289349 (PMC10414650; doi:10.1371/journal.pone.0289349)
Supplement: S6 Appendix — (PDF) [file pone.0289349.s006.pdf]

S6 Appendix. Questionnaire survey and field research.

(Questions, results, and findings)

The questionnaire survey is oriented to investigated public perception and preference of Chinese residents on remanufacturing and remanufactured products. Questionnaires are distributed to collect raw data in the Yangtze River Delta, which is one of the largest delta areas in China comprised of Zhejiang Province, Jiangsu Province and Shanghai City etc. Due to the high density of population and prosperous manufacturing industry in this region, more than 80% pilot enterprises of remanufacturing in China were established. Yangtze River Delta is therefore frequently regarded as one typical area for empirical researches in the field of remanufacturing industry by questionnaires. During about five months of investigation, we distributed 700 questionnaires and received 611 valid ones.

Our research team spent more than 20 days in each city of Zhejiang and Jiangsu province, to distribute questionnaires in campuses, markets and public squares, etc. In the capitals of Zhejiang and Jiangsu province, we collected 16.2% and 15.8% questionnaires in Hangzhou and Nanjing respectively. Shanghai which is one of largest municipalities in China has provided 13.5% questionnaires for our research team. In order to further improve the validity of questionnaire samples, we also distributed questionnaires in other small- and medium-sized cities. In Ningbo, Taizhou and Huzhou of Zhejiang province, for instance, we collected 11.8%, 9.4% and 8.8% of total questionnaires respectively. 9.5%, 8.2% and 7.8% of valid questionnaires are retrieved in Wuxi, Suzhou and Changzhou of Jiangsu Province.

Based on questionnaire design, we classify the questionnaire consisting of 20 questions into five groups, presenting as "Basic information of the respondents", "Promulgation of promotional channels of the remanufactured products", "Purchase experience of residents for remanufactured products", "public awareness on purchase channels of remanufactured products", and "public awareness of the necessity to popularize the remanufactured products in China".

The questions and results are provided as follows.

Questions and results

1. Gender.

Options	Results	Proportions
Male	323	52.86%
Female	288	47.14%
Valid questionnaires of this question	611	

2. Age.

Options	Results	Proportions
Under 20	81	13.26%
20-29	251	41.08%
30-39	108	17.68%
40-49	135	22.09%
50-59	30	4.91%
Above 60	6	0.98%
Valid questionnaires of this question	611	

3. Disposable personal income per month.

Options	Results	Proportions
Under 1500	163	26.68%
1500-5000	229	37.48%
5000-10000	132	21.6%
10000-15000	38	6.22%
Above 15000	49	8.02%
Valid questionnaires of this question	611	

4. Education Background.

Options	Results	Proportions
Junior high school graduation	58	9.49%
High school graduation	154	25.2%
Bachelor degree	274	44.84%
Master degree or above	125	20.46%
Valid questionnaires of this question	611	

5. Career.

Options	Results	Proportions
Student	210	34.37%
Service industry	86	14.08%
Agriculture	11	1.8%
Industry	77	12.6%
Commercial industry	84	13.75%
Public office (including school)	64	10.47%
Retired	13	2.13%
Other	66	10.8%
Valid questionnaires of this question	611	

6. Purchase experience of remanufactured products.

Options	Results	Proportions
Have purchased	186	30.44%
Have not purchased	425	69.56%
Valid questionnaires of this question	611	

7. Cognitive level of remanufactured products.

Options	Results	Proportions
Heard	326	53.36%
Not Heard	285	46.64%
Valid questionnaires of this question	611	

8. Information channels of remanufactured products (Multiple choices).

Options	Results	Proportions
Acquaintance	115	35.28%
After-sale service center	41	12.58%
Retailer	46	14.11%
Internet	198	60.74%
Newspaper	68	20.86%
Government	18	5.52%
TV & broadcast	92	28.22%
Other	26	7.98%
Valid questionnaires of this question	326	

9. Preferred responsible entities for promoting remanufactured products (Multiple choices).

Options	Results	Proportions
Government	171	52.45%
Original manufacturers	107	32.82%
Retailers	43	13.19%
Remanufacturers	138	42.33%
Distributors	24	7.36%

Experts	88	26.99%
Non-government organizations	20	6.13%
Acquaintances	79	24.23%
Other	13	3.99%
Valid questionnaires of this question	326	

10. Willingness to buy a remanufactured product at half price compared with a new one.

Options	Results	Proportions
Willing	442	72.34%
Not willing	169	27.66%
Valid questionnaires of this question	611	

11. Worries in purchasing remanufactured products (Multiple choices).

Options	Results	Proportions
Potential safety hazard due to substandard quality	119	70.41%
Poorer quality and performance than new products	99	58.58%
Potential economic loss due to substandard quality	78	46.15%
Inadequate after-sale service	73	43.2%
Worries in having fake goods mixed among the remanufactured products	86	50.89%
Other	21	12.43%
Valid questionnaires of this question	169	

12. Preferred manufacturers of remanufactured products (Multiple choices).

Options	Results	Proportions
Original manufacturers	423	69.23%
Third-party remanufacturers that qualified with national certifications	426	69.72%
General shops	15	2.45%
Similar manufacturers	60	9.82%
Else	20	3.27%
Valid questionnaires of this question	611	

13. Pre-purchase considerations in purchasing remanufactured products (Multiple choices).

Options	Results	Proportions
Appearance	164	26.84%
Performance	478	78.23%
Quality	516	84.45%
Price	345	56.46%
After-sale service	399	65.3%
Sales channel	92	15.06%
Popularity	69	11.29%
Production qualification	129	21.11%
Environmental level (Sustainability, Recyclability)	192	31.42%
Other	12	1.96%
Valid questionnaires of this question	611	

14. Cognitive level of purchase channels of remanufactured products

Options	Results	Proportions
Heard	100	16.37%
Not heard	511	83.63%
Valid questionnaires of this question	611	

15. Preferred purchase channels of remanufactured products (Multiple choices).

Options	Results	Proportions
Original manufacturer	64	64%
Supermarket	26	26%
Online channel	40	40%
Third party remanufacturer	53	53%
Export	12	12%
Other	1	1%
Valid questionnaires of this question	100	

16. Cognitive level of propagandizing remanufactured products

Options	Results	Proportions
Heard	179	29.3%
Nor heard	432	70.7%
Valid questionnaires of this question	611	

17. Cognitive level of remanufacturing laws and regulations, for example: swap the old for remanufacturing.

Options	Results	Proportions
Heard	255	41.73%
Not heard	356	58.27%
Valid questionnaires of this question	611	

18. Necessity of promoting remanufactured products that are environmentally friendly

Options	Results	Proportions
Necessary	576	94.27%
Not necessary	35	5.73%
Valid questionnaires of this question	611	

19. Responsibility entities in promoting remanufactured products (Multiple choices).

Options	Results	Proportions
Government	455	74.47%
Manufacturer	340	55.65%
Remanufacturer	398	65.14%
Retailer	168	27.5%
Consumer	80	13.09%
Other	8	1.31%
Valid questionnaires of this question	611	

20. Preferred Measures to promote remanufactured products (Multiple choices).

Options	Results	Proportions
Laws and Regulations	508	83.14%
Community popularization	269	44.03%
Expand procurement scale of remanufactured products by government	192	31.42%
Government subsidy for remanufactured product	239	39.12%
Advertisement and Discount activities by firms	237	38.79%
Acquaintance's publicity	185	30.28%
Other	24	3.93%
Valid questionnaires of this question	611	

In addition to questionnaire survey, filed research was also conducted via interviews to document awareness of certain people on remanufactured products. During the filed research, our research team visited campuses, shops, squares and other public places in each surveyed city to interview with people who have different occupations such as the teaching and administrative staff, salesmen and managers in the shops, students and workmen etc.

Some example questions of interview are introduced as follows:

- *Have you ever heard of remanufactured products? Do you know the concepts of it?*
- *Which attribute of remanufactured products do you care most? For example: price, capacity etc.*
- *Which information channels help you to understand remanufactured products?*
- *Have you ever heard some national policies related to remanufacturing such as 'Swap the Old for Remanufacturing'?*
- *Which one should be in charge of responsibility of environmental protection?*

We visited Good Friend Precision Machinery to accumulate information regarding its basic information, remanufacturing scale, operations, and subsidy provided by government. Good Friend Precision Machinery is a specialized enterprise in the manufacture and export of numerical control machines. It has also been engaged in remanufacturing waste machine tools for years. It collects and remanufactures end-of-life products when a client enterprise sends the order for remanufacturing.

Survey findings

This questionnaire survey investigates the public awareness of Chinese residents for remanufactured products, main findings and problems are illustrated as follows.

- Weak awareness and lack of propaganda on remanufactured products

Overall, 46.6% respondents replied that they never heard about the remanufactured products, and only 30.44% have purchased remanufactured products. As for the related policies such as *Swap* the *old for remanufacturing*, *Sample pilot enterprises of remanufacturing* and *The List of Remanufactured Products*, only 29.3% respondents were aware of them. It is obvious that public recognition of remanufactured products and other environmentally-friendly goods is comparatively weak in current China. Worse still, relevant publicity of remanufactured products is far from enough; the policies and legislation in the field of remanufacturing have aroused only slight public attention and often lag in execution.

- Deficient legislation on remanufacturing and lacking dissemination of policies

For the sake of promoting the remanufacturing industry along with its products, enactment of laws is regarded as one indispensable measure. In practice, Chinese government published *The Circular Economy Promotion Law* in August of 2008, planning to facilitate and regulate remanufacturing in car parts, machinery, etc., which requires all the products to meet national quality standards, and to be marked clearly with remanufacturing labels. While such laws and regulations have been issued and have indeed worked to the advancement of remanufacturing industry and remanufactured products, they are generally legislated at a comprehensive level, applying to various trades and professions covering remanufacturing and related occupations. In this sense, specific laws targeting the remanufacturing industry have been absent so far. In accordance with reality, our statistical results show that 70.7% respondents are unaware of policies such as the *Swap the Old for Remanufacturing* which is the most noticeable legislation regarding remanufacturing, published in 2013. It stipulated that subsidies should be provided for remanufacturing enterprises from the government, and customers are entitled to buy remanufactured products at some discount, 15% on average, if used products of the same kind are collected. Existing supporting legislation in like manner is supposed to be promulgated and popularized for a better understanding of improved customer benefits.

- Poor sales performance and weak enthusiasm of customers

Based on our research results, about one-third (30.4%, 186 of 611) of respondents reflected that they have bought remanufactured products. Most of them, 34.4% and 28.5% of the 186 respondents, stated that they purchased the remanufactured products via original manufacturers and from third-party remanufacturing enterprises. Statistical results are evident that most remanufactured products in current China are collected, scrapped and marketed by original manufacturers, manifesting as an OEM remanufacturing mode. Third-party enterprises of remanufacturing are sometimes allowed to process and distribute remanufactured products after authorization, also generating a considerable number of remanufactured products. In addition, 72.3% respondents expressed their willingness to buy remanufactured products if the price is 50–60% of the original price, clarifying current pricing of remanufactured commodities with a 20–30% reduction of newly-made ones as an undesirable outcome, and discouraging potential

customers to make purchases at that price.

Generally, public awareness on emerging products with environment-favorable attributes was relatively weak. Relatively few residents are possessed with a sound knowledge of remanufactured products due to deficient information from governments and enterprises. Worse still, nearly 70% of them have not purchased remanufactured products despite 72.3% of respondents who express their willingness to pay. Fortunately, most respondents believe it will be necessary and important to popularize environmentally-friendly products, and both the government and enterprises are supposed to be in charge of the duty to protect and ameliorate environment pollution.

The field research of Good Friend Precision Machinery provides significant information of remanufacturing management and operations of some remanufacturers in current development stage of Chinese remanufacturing industry.

We note that Good Friend Precision Machinery is a small-scale firm with limited production capacity, capital and technology to fulfil remanufacturing orders. Besides, output of its remanufactured machine tools is limited, and practically constant despite demand fluctuation and consumer preference variance due to limited production capacity and technology.

The regulation of "Swap the old for remanufacturing" executed by Chinese government stipulates that subsidies should be offered for remanufactured products regarding automobile engines, transmission cases, and large machine tools, among others. The government provides 10% of the replacement value for the remanufactured products possessed with domestic certifications and qualified for sale, while 2000 CNY (~$310) at most would be allocated for a unit of remanufactured product. In the case that Good Friend Precision Machinery acts as the remanufacturer, unit subsidy of 1750 CNY (~$271) is offered.
