# Supplementary material for: A remanufacturing supply chain network with differentiated new and remanufactured products considering consumer preference, production capacity constraint and government regulation
Source: PLoS One. 2023 Aug 10;18(8):e0289349. doi: 10.1371/journal.pone.0289349 (PMC10414650; doi:10.1371/journal.pone.0289349)
Supplement: S7 Appendix — (PDF) [file pone.0289349.s007.pdf]

S6 Appendix. Algorithm.

The logarithmic-quadratic proximal prediction-correction (LQP-PC) method is employed for the computation of the solutions to solve the variational inequality problem (20).

Denote F_1, FR_1, F_2, FR_2, F_3, F_4, F_5, FR_5, F_6, FR_6 as row vectors, which are expresses as:

$$F_1 = \sum_{i=1}^{m} \left[\frac{\partial c_i^{N*}}{\partial q_i^N} + \frac{\partial f_i^{N*}}{\partial q_i^N}, i = 1, \cdots, m \right] \in R^m \tag{S6.1}$$

$$FR_1 = \sum_{k=1}^{o} \left[\frac{\partial c_k^{R*}}{\partial q_k^R} - \frac{\partial s_k^{R*}}{\partial q_k^R}, k = 1, \cdots, o \right] \in R^o \tag{S6.2}$$

$$F_2 = \sum_{i=1}^{m}\sum_{j=1}^{n} \left[\frac{\partial w_{ij}^*}{\partial q_{ij}^N} + \frac{\partial w_j^*}{\partial q_{ij}^N}, i = 1, \cdots, m; j = 1, \cdots, n \right] \in R^{mn} \tag{S6.3}$$

$$FR_2 = \sum_{i=1}^{m}\sum_{j=1}^{n} \left[\frac{\partial w_{ij}^*}{\partial q_{ij}^R} + \frac{\partial w_j^*}{\partial q_{ij}^R}, i = 1, \cdots, m; j = 1, \cdots, n \right] \in R^{mn} \tag{S6.4}$$

$$F_3 = \sum_{k=1}^{o}\sum_{i=1}^{m} \left[\frac{\partial w_{ki}^*}{\partial q_{ki}^R}, k = 1, \cdots, o; i = 1, \cdots, m \right] \in R^{om} \tag{S6.5}$$

$$F_4 = \sum_{k=1}^{o} \left[\alpha^* + \frac{\partial w_k^*}{\partial q_k^E}, k = 1, \cdots, o \right] \in R^k \tag{S6.6}$$

$$F_5 = \sum_{j=1}^{n} \left[p_j^{N*} \sigma_j^{N*} - p_j^{N*}, j = 1, \cdots, n \right] \in R^n \tag{S6.7}$$

$$FR_5 = \sum_{j=1}^{n} \left[p_j^{R*} \sigma_j^{R*} - p_j^{R*}, j = 1, \cdots, n \right] \in R^n \tag{S6.8}$$

$$F_6 = \sum_{j=1}^{n} \left[q_j^{N*} - E\left[d_j^R \right], j = 1, \cdots, n \right] \in R^n \tag{S6.9}$$

$$FR_6 = \sum_{j=1}^{n} \left[q_j^{R*} - E\left[d_j^R \right], j = 1, \cdots, n \right] \in R^n \tag{S6.10}$$

We now state the network equilibrium problem as: determine $\left(Q_m^{N*}, Q_o^{R*}, Q_{mn}^{N*}, Q_{mn}^{R*}, Q_{om}^{R*}, Q_o^{E*}, Q_n^{N*}, Q_n^{R*}, \; P_n^{N*}, P_n^{R*}, \lambda^*, \gamma^*, \mu^*, \eta^*, \varepsilon^*, \beta^*, \xi^*\right) \in K$, satisfying

$$\begin{aligned} &F_1 \times \left(Q_m^N - Q_m^{N*}\right) + FR_1 \times \left(Q_o^R - Q_o^{R*}\right) + F_2 \times \left(Q_{mn}^N - Q_{mn}^{N*}\right) + FR_2 \times \left(Q_{mn}^R - Q_{mn}^{R*}\right) + \\ &F_3 \times \left(Q_{om}^R - Q_{om}^{R*}\right) + F_4 \times \left(Q_o^E - Q_o^{E*}\right) + F_5 \times \left(Q_n^N - Q_n^{N*}\right) + FR_5 \times \left(Q_n^R - Q_n^{R*}\right) + \\ &F_6 \times \left(P_n^N - P_n^{N*}\right) + FR_6 \times \left(P_n^R - P_n^{R*}\right) \geq 0 \end{aligned} \tag{S6.11}$$

where the feasible set K is

$$K = \left\{ \begin{array}{l|l} Q_m^N \in R_+^m & \\ Q_o^R \in R_+^o & A_1 Q_m^N \le \bar{q} \\ Q_{mn}^N \in R_+^{mn} & A_2 Q_{mn}^N \le Q_m^N \\ Q_{mn}^R \in R_+^{mn} & A_2 Q_{mn}^R \le A_3 Q_{om}^R \\ Q_{om}^R \in R_+^{om} & Q_n^N = A_4 Q_{mn}^N \\ Q_o^E \in R_+^o & Q_n^R = A_4 Q_{mn}^R \\ Q_n^N \in R_+^n & Q_o^E \le A_5 Q_n^N \\ Q_n^R \in R_+^n & A_6 Q_{om}^R \le Q_o^R \\ P_n^N \in R_+^n & Q_o^R \le A_7 Q_o^E \\ P_n^R \in R_+^n & \end{array} \right\} \tag{S6.12}$$

where $A_1 = E_{m\times m}$, $A_5 = E_{n\times n}$, $A_7 = E_{o\times o}$ are identity matrixes. $A_2 \in R^{m\times mn}$, $A_3 \in R^{m\times om}$, $A_4 \in R^{n\times mn}$, $A_6 \in R^{o\times om}$ are block matrixes expressed as:

$$A_2 = \begin{bmatrix} I_1 & 0 & \cdots & 0 \\ 0 & I_2 & \cdots & 0 \\ \vdots & \vdots & \vdots & \vdots \\ 0 & 0 & \ldots & I_m \end{bmatrix}_{m\times mn} \tag{S6.13}$$

$$A_3 = \begin{bmatrix} J_1 & J_1 & \cdots & J_1 \\ J_2 & J_2 & \cdots & J_2 \\ \vdots & \vdots & \vdots & \vdots \\ J_m & J_m & \ldots & J_m \end{bmatrix}_{m\times om} \tag{S6.14}$$

$$A_4 = \begin{bmatrix} K_1 & K_1 & \cdots & K_1 \\ K_2 & K_2 & \cdots & K_2 \\ \vdots & \vdots & \vdots & \vdots \\ K_m & K_m & \ldots & K_m \end{bmatrix}_{n\times mn} \tag{S6.15}$$

$$A_6 = \begin{bmatrix} N_1 & 0 & \cdots & 0 \\ 0 & N_2 & \cdots & 0 \\ \vdots & \vdots & \vdots & \vdots \\ 0 & 0 & \ldots & N_o \end{bmatrix}_{o\times om} \tag{S6.16}$$

In (S6.13)-(S6.16), $I_i = (1,\cdots,1) \in R^m$, $i = 1,\cdots,m$ and $N_k = (1,\cdots,1) \in R^o$, $k = 1,\cdots,o$ are row vectors whose elements are 1. $J_i = (0,\cdots,1,\cdots 0) \in R^m$, $i = 1,\cdots,m$ and $K_j = (0,\cdots,1,\cdots 0) \in R^n$, $j = 1,\cdots,n$ are row vectors whose j-th element is 1.

All column vectors of variables $\left(Q_m^N, Q_n^R, Q_{mn}^N, Q_{mn}^R, Q_{om}^R, Q_o^E, Q_n^N, Q_n^R, P_n^N, P_n^R\right)$ constitute the column vector *x*, and all constants in the constraints formulate the column vector *b*. We set each pair of *x* and the matrix, satisfying $A^T x \le b$. *y* is denoted as the column vector formulated by all Lagrange multipliers, and $f(x)$ is a column vector formed by $\left(F_1^T, FR_1^T, F_2^T, FR_2^T, F_3^T F_4^T, F_5^T, FR_5^T, F_6^T, FR_6^T\right)$.

The computational procedure is listed below:

Step 0: Initialization

Initialize parameters and initial solutions. Let $\beta^0 = 1$, $\upsilon = 1$, η=0.9, μ=0.1, γ=1.8, ε=10^{-8}, $u^0 = \left(x^0, y^0\right)$, l=0.

Step 1: Termination criteria

Denote the error vector as

$$e\left(u^l\right) = \begin{pmatrix} Q_m^N - P_{R_+^m}\left\{Q_m^N - \left(F_1 + A_1\lambda - A_1\gamma\right)\right\} \\ Q_o^R - P_{R_+^o}\left\{Q_o^R - \left(FR_1 - A_7\beta + A_7\rho\right)\right\} \\ Q_{mn}^N - P_{R_+^{mn}}\left\{Q_{mn}^N - \left(F_2 + A_2\gamma - A_3\eta\right)\right\} \\ Q_{mn}^R - P_{R_+^{mn}}\left\{Q_{mn}^R - \left(FR_2 + A_2\mu - A_3\varepsilon\right)\right\} \\ Q_{om}^R - P_{R_+^{om}}\left\{Q_{om}^R - \left(F_3 - A_3\mu + A_6\beta\right)\right\} \\ Q_o^E - P_{R_+^o}\left\{Q_o^E - \left(F_4 - A_7\rho + \xi\right)\right\} \\ Q_n^N - P_{R_+^n}\left\{Q_n^N - \left(F_5 + A_5\eta\right)\right\} \\ Q_n^R - P_{R_+^n}\left\{Q_n^R - \left(FR_5 + A_5\varepsilon\right)\right\} \\ P_n^N - P_{R_+^n}\left\{P_n^N - F_6\right\} \\ P_n^R - P_{R_+^n}\left\{P_n^R - FR_6\right\} \\ \lambda - P_{R_+^m}\left\{\lambda + A_1 Q_m^N - A_1\bar{q}\right\} \\ \gamma - P_{R_+^{mn}}\left\{\gamma + A_2 Q_{mn}^N - A_1 Q_m^N\right\} \\ \mu - P_{R_+^{mn}}\left\{\mu + A_2 Q_o^R - A_3 Q_{mn}^N\right\} \\ \eta - P_{R_+^{mn}}\left\{\eta + Q_n^N - A_4 Q_{mn}^N\right\} \\ \varepsilon - P_{R_+^{mn}}\left\{\varepsilon + Q_n^R - A_4 Q_{mn}^R\right\} \\ \beta - P_{R_+^{no}}\left\{\beta + Q_o^E - A_5 Q_n^N\right\} \\ \xi - P_{R_+^n}\left\{\xi + Q_o^R - A_7 Q_o^E\right\} \end{pmatrix} \quad \text{(S6.17)}$$

where $P_Y(y)$ is projection of vector y on to the convex set Y.

If $\left\| e\left(u^l\right)\right\|_\infty < \varepsilon$, then stop; else, go to **Step 2**.

Step 2: Prediction error value $\tilde{u}^k = \left(\tilde{x}^k, \tilde{y}^k\right)^T$

Step 2.1: Compute r^l in accordance to step length β^l.

$$\tilde{y}^l = P_y\left[y^l + \left(M^T x^l - b\right)\beta^l / \upsilon\right] \tag{S6.18}$$

$$s = (1-\mu)x^l - \beta^l\left(f\left(x^l\right) + M\tilde{y}^l\right) \tag{S6.19}$$

$$\tilde{x}_i^l = \left(s_i + \sqrt{\left(s_i\right)^2 + 4\mu\left(x_i^l\right)^2}\right)\Big/2 \tag{S6.20}$$

$$\pi_1^l = x^l - \tilde{x}^l, \quad \pi_2^l = y^l - \tilde{y}^l \tag{S6.21}$$

$$\xi_1^l = \beta^l\left(f\left(\tilde{x}^l\right) - f\left(x^l\right)\right), \quad \xi_2^l = \beta^l M^T\left(x^l - \tilde{x}^l\right) \tag{S6.22}$$

$$r^l = \sqrt{\frac{\upsilon\left\|\xi_1^l\right\|^2 + (1+\mu)\left\|\xi_2^l\right\|^2}{\upsilon\left(1-\mu^2\right)\left\|\pi_1^l\right\|^2 + \upsilon^2(1-\mu)\left\|\pi_2^l\right\|^2}} \tag{S6.23}$$

Step 2.2: if $r^l > \eta$, then $\beta^l = \beta^l \times 0.8/r^l$ and return to Step 2.1; else, go to Step 3.

Step 3: Adaption for step length β^l **and parameter** υ.

$$\beta^{l+1} = \begin{cases} \beta^l \times 0.7/r^l, & r^l \le 0.5 \\ \beta^l, & else \end{cases} \tag{S6.24}$$

$$\upsilon = \begin{cases} 0.5\upsilon, & \left\|\xi_1^l\right\|/\sqrt{1+\mu} > 4\left\|\xi_2^l\right\|/\sqrt{\upsilon} \\ 2\upsilon, & 4\left\|\xi_1^l\right\|/\sqrt{1+\mu} < \left\|\xi_2^l\right\|/\sqrt{\upsilon} \\ \upsilon, & else \end{cases} \tag{S6.25}$$

Step 4: The calibrated step length

$$\alpha^l = \gamma\alpha^{l*}\beta^l(1-\mu)/(1+\mu) \tag{S6.26}$$

where

$\alpha^{l*} = \left[\left(\pi_1^l + \xi_1^l\right)\pi_1^l + \left(\upsilon\pi_2^l + \xi_2^l\right)\pi_2^l\right] \Big/ \Big\{\left[(1+\mu)\pi_1^l + \xi_1^l\right]^T \left[\pi_1^l + (1+\mu)^{-1}\xi_1^l\right] + \left(\upsilon\pi_2^l + \xi_2^l\right)^T \left(\pi_2^l +\right.$

$\left.\upsilon^{-1}\xi_2^l\right)\Big\}$.

Step 5: The error value $u^{l+1} = \left(x^{l+1}, y^{l+1}\right)^T$

$$s = (1-\mu)x^l - \alpha^l\left(f\left(\tilde{x}^l\right) + M\tilde{y}^l\right) \quad \text{(S6.27)}$$

$$x_i^{l+1} = \left(s_i + \sqrt{(s_i)^2 + 4\mu\left(x_i^l\right)^2}\right) \Big/ 2 \quad \text{(S6.28)}$$

$$y^{l+1} = P_y\left[y^l + \left(M^T\tilde{x}^l - b\right)\alpha^l/\upsilon\right] \quad \text{(S6.29)}$$

Let $l = l+1$, and then go to Step 1.
